# Plant hairy roots enable high throughput identification of antimicrobials against *Candidatus* Liberibacter spp.

**DOI:** 10.1038/s41467-020-19631-x

**Published:** 2020-11-16

**Authors:** Sonia Irigoyen, Manikandan Ramasamy, Shankar Pant, Prakash Niraula, Renesh Bedre, Meena Gurung, Denise Rossi, Corinne Laughlin, Zachary Gorman, Diann Achor, Amit Levy, Michael V. Kolomiets, Mamoudou Sétamou, Ismael E. Badillo-Vargas, Carlos A. Avila, Michael S. Irey, Kranthi K. Mandadi

**Affiliations:** 1 Texas A&M AgriLife Research and Extension Center, Weslaco, TX USA; 2 Department of Plant Pathology and Microbiology, Texas A&M University, College Station, TX USA; 3 Citrus Research and Education Center, University of Florida, Lake Alfred, FL USA; 4 Department of Plant Pathology, University of Florida, Gainesville, FL USA; 5 Texas A&M University-Kingsville, Citrus Center, Weslaco, TX USA; 6 Department of Entomology, Texas A&M University, College Station, TX USA; 7 Department of Horticultural Sciences, Texas A&M University, College Station, TX USA; 8 Southern Gardens Citrus, Clewiston, FL USA; 9 Present Address: Agricultural Research Service, US Department of Agriculture, Stillwater, OK USA

**Keywords:** High-throughput screening, Agricultural genetics, Biotic

## Abstract

A major bottleneck in identifying therapies to control citrus greening and other devastating plant diseases caused by fastidious pathogens is our inability to culture the pathogens in defined media or axenic cultures. As such, conventional approaches for antimicrobial evaluation (genetic or chemical) rely on time-consuming, low-throughput and inherently variable whole-plant assays. Here, we report that plant hairy roots support the growth of fastidious pathogens like *Candidatus* Liberibacter spp., the presumptive causal agents of citrus greening, potato zebra chip and tomato vein greening diseases. Importantly, we leverage the microbial hairy roots for rapid, reproducible efficacy screening of multiple therapies. We identify six antimicrobial peptides, two plant immune regulators and eight chemicals which inhibit *Candidatus* Liberibacter spp. in plant tissues. The antimicrobials, either singly or in combination, can be used as near- and long-term therapies to control citrus greening, potato zebra chip and tomato vein greening diseases.

## Abstract (web summary)

The putative causal agent of citrus greening *Candidatus* Liberibacter asiaticus (*C*Las) cannot be cultured, which hampers finding new therapies to control this devastating disease. Here, the authors show that hairy roots support *C*Las propagation and enable high throughput antimicrobial screening.

## Introduction

Fastidious and obligate phytopathogens cause devastating diseases in several food and commodity crops: for example, the phloem-limited bacteria *Candidatus* Liberibacter spp. cause severe yield losses in the Solanaceae [potato (*Solanum tuberosum* L.), tomato (*S. lycopersicum* L.), pepper (*Capsicum annuum* L.), and tobacco (*Nicotiana tabacum* L.)] and Rutaceae (*Citrus* spp.) families^1^. Potato zebra chip (ZC) disease, putatively caused by *Candidatus* Liberibacter solanacearum (*C*Lso), is transmitted by an insect vector, the potato psyllid [*Bactericera cockerelli* (Šulc); Hemiptera: Triozidae]^2^. Since its discovery in 1994, ZC has been documented in several commercial potato-growing regions of the United States, Mexico, Central America, Australia, and New Zealand, causing yield losses of up to 94%^3,4^. Similarly, citrus greening or huanglongbing (HLB), putatively caused by *Candidatus* Liberibacter asiaticus (*C*Las), is the most devastating disease threatening citrus production worldwide; its insect vector is the Asian citrus psyllid (*Diaphorina citri* Kuwayama [Hemiptera: Liviidae])^5^. Between 2006 and 2011, HLB caused losses of more than US$4.5 billion in the US state of Florida alone^6^. *C*Las and *C*Lso share some commonalities, such as their unculturable nature, genomic attributes, and lifestyles as psyllid-vectored and phloem-limited bacteria^7–10^. These and other diseases caused by fastidious plant pathogens are major threats to global agriculture and food security.

A challenge in identifying therapies against *C*Las and *C*Lso is our inability to culture or maintain them outside their plant and insect hosts. Over 99% of all microorganisms are estimated to be unculturable in the laboratory^11^. Attempts have been made to customize artificial growth media and culture conditions to culture such microbes, including specialized modifications to growth medium composition and growth conditions tailored to the particular microbe based on what we know about its natural growth environment (e.g., temperature, pH, incubation time, inoculum size, and CO_2_/O_2_ ratio), coculturing with other microbes, and growth in biofilms or other simulated environments^11–21^. Although these approaches have allowed some success in mono- or cocultures as means to propagate fastidious pathogens but they have provided little contribution to downstream applications such as the screening of antimicrobials against target pathogens, and therefore are not broadly utilized. Because of the constraints that exist in culturing fastidious pathogens in defined media and evaluating potential therapies, we explored alternative strategies.

Hairy roots are neoplastic growth that forms on plant tissues after wounding and infection with the soil bacterium *Rhizobium rhizogenes* (previously *Agrobacterium rhizogenes*)^22,23^. *R. rhizogenes* introduces its root-inducing transfer DNA (Ri T-DNA), encoding the *root locus* (*rolA*, *rolB*, *rolC*, and *rolD*) genes, into the plant genome. Expression of *rol* genes in planta modulates auxin homeostasis and induce hairy root initiation and proliferation in vitro; thus, hairy root cultures do not require the addition of phytohormones. Hairy roots are anatomically and metabolically similar to normal roots, and possess intact xylem and phloem vasculature connected to the source explant^24–26^. They constitute a valuable tool in plant functional biology, biotechnology, metabolic engineering, molecular pharming, and studies of root–rhizosphere interactions^26–29^. Classical microbiological techniques developed early in the 19th century, cultured animal and mammalian viruses in host cells, tissues, and embryonated eggs^30–36^. In a similar manner, we hypothesized that plant hairy roots could be suitable for propagating fastidious pathogens.

In this work, we show that hairy roots support the accumulation of fastidious plant bacteria, *Candidatus* Liberibacter spp. Furthermore, using the microbial hairy roots, we conduct antimicrobial screening leading to the discovery of multiple genetic and chemical therapies that effectively suppress *Candidatus* Liberibacter spp. in plant tissues.

## Results

### Hairy roots support propagation of fastidious pathogens

**Fig. 1:**
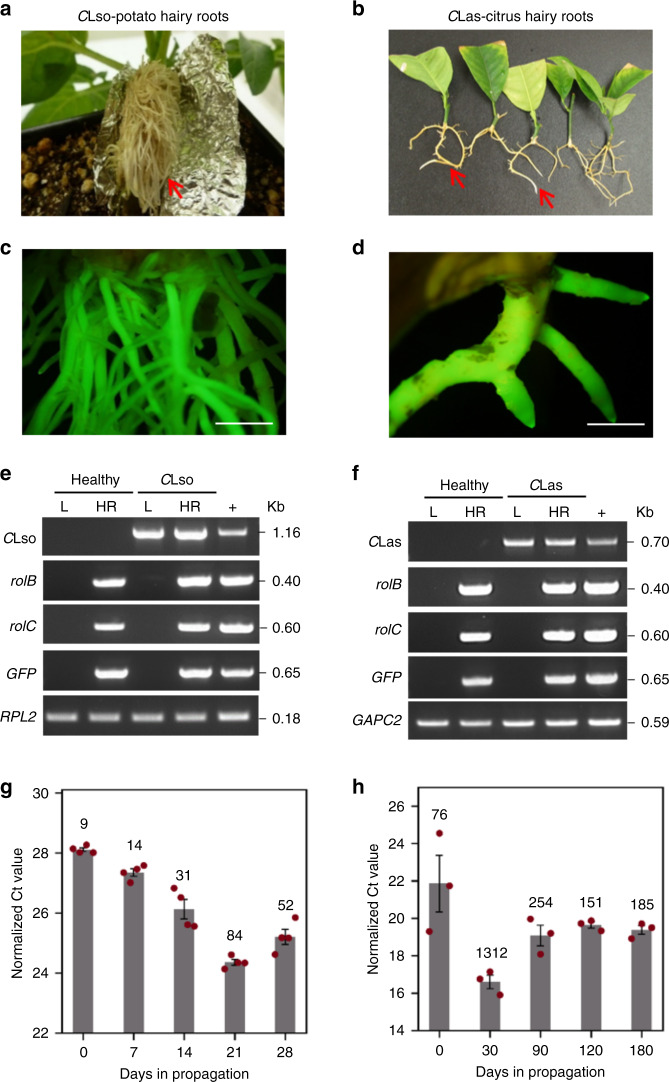
*Candidatus* Liberibacter solanacearum and *Candidatus* Liberibacter asiaticus in potato and citrus hairy roots. **a**, **b** Hairy roots (indicated by arrows) from *Candidatus* Liberibacter solanacearum (*C*Lso)-infected potato (**a**) and *Candidatus* Liberibacter asiaticus (*C*Las)-infected citrus explants (**b**). **c**, **d** Visual confirmation of GFP expression in hairy roots by fluorescence microscopy. Scale bars, 1 cm. **e**, **f** Detection of *C*Lso and *C*Las in the hairy roots by PCR amplification of diagnostic markers specific to *C*Lso (16S rDNA) and *C*Las (rplk04/J5 and RNR). *GFP*, *rolB*, and *rolC* encoded on the Ti and Ri plasmids, respectively, and co-transformed into the hairy roots, were used as additional markers for hairy root authenticity; *RPL2* and *GAPC2* are endogenous potato and citrus genes, respectively, used as genomic DNA controls for PCR. “L” and “HR” indicate leaf and hairy root samples, respectively. “+” indicates positive controls used for the respective PCR amplifications. **g**, **h** Temporal growth curves of *C*Lso and *C*Las in the hairy roots. The Ct values of *C*Lso and *C*Las in hairy root samples collected at different days in propagation are plotted, whereas the approximate genome equivalents (GE) per nanogram of root genomic DNA are indicated above the bar graph columns. Error bars represent ± standard error of mean (*n* = 4 and *n* = 3 for **g** and **h**, respectively). Uncropped raw agarose gel images used to prepare **e** and **f** are presented in Supplementary Fig. 10. Source data underlying Fig. 1g, h are provided as a Source Data file.

Since *Candidatus* Liberibacter spp. (*C*Lso and *C*Las) are phloem-limited pathogens, we tested whether plant hairy roots, which possess intact vasculature^24–26^, could sustain their growth. Hairy roots are anatomically and metabolically similar to normal roots and can be induced in a variety of plants using *R. rhizogenes*^24–26^. To test our hypothesis, we generated ex vivo and in planta aerial hairy roots from *C*Lso- and *C*Las-infected potato, tomato and citrus explant tissues (Fig. 1a, b and Supplementary Fig. 1). We used *R. rhizogenes* harboring a binary T-DNA vector with a green fluorescent protein (GFP) marker to induce hairy root growth. Fluorescence microscopy analysis confirmed the production and authenticity of the transgenic hairy roots, which were distinguished from the adventitious roots by their GFP signal (Fig. 1c, d). The transformation efficiency of the hairy root induction, defined by the percentage of GFP-positive hairy roots among all roots generated, was ~70–90% in potato and tomato and ~50–70% in citrus.

The genes, *rolB* and *rolC*, encoded by the Ri T-DNA of *R. rhizogenes*, were amplified using a polymerase chain reaction (PCR) and used as additional markers to confirm the transformation of the hairy roots (Fig. 1e, f). PCR amplification using diagnostic markers specific to *C*Lso (16S rDNA)^37,38^ and *C*Las (A2/J5 and RNR)^39,40^ readily detected these pathogens in the infected potato and citrus hairy roots, but not in the uninfected hairy roots (Fig. 1e, f). Similar results were obtained with *C*Lso in a second host, tomato (Supplementary Fig. 3). The identity of *C*Lso and *C*Las in the hairy roots was confirmed by Sanger sequencing and Basic Local Alignment Search Tool (BLAST) analysis of the respective PCR amplicons for similarity to the NCBI nucleotide sequence database (Supplementary Fig. 8). Copy number and bacterial growth curve estimation by quantitative (q) PCR analysis further showed that *C*Lso and *C*Las could be maintained in the potato and citrus hairy roots for at least 28 and 120 days, respectively (Fig. 1g, h).

Next, we performed transmission electron microscopy (TEM) to visualize *C*Lso/*C*Las and probe the anatomy of the hairy roots. Unlike PCR/Sanger sequencing or immuno-labeling approaches, TEM alone is not a confirmatory test to identify pathogens. Unfortunately, immuno-labeling is technically challenging to perform for *C*Lso/*C*Las detection, primarily due to limitations of existing antibodies (only available for *C*Las) and the low-titers and highly variable nature of *C*Lso/*C*Las accumulation in infected plant tissues^41^. Thus far, *C*Las immuno-labeling was primarily successful in insect-vector^42^. Nevertheless, our TEM analysis showed multiple rounds- and bacilliform-shaped bacteria-like cells in the infected potato and citrus hairy roots, but not in healthy hairy roots (Supplementary Fig. 9), along with notable symptoms of vascular deterioration and collapse, presumably caused by *C*Lso/*C*Las^43,44^. The morphology and the vascular degeneration phenotypes were reminiscent of those reported for *C*Las-infected citrus^43,44^. Furthermore, the lack of other microbes in healthy tissues suggests that the normal microbiome is well-below the detection limits of TEM hence the bacteria-like cells observed in the infected tissues are associated with *C*Lso/*C*Las infection. Combined with the Sanger-sequencing confirmatory analysis, our results sufficiently demonstrate the presence of *C*Lso/*C*Las in the hairy roots and suggest that the *C*Lso/*C*Las can be maintained in plant hairy roots like normal infected plant roots.

### Genetic evaluation using microbial hairy roots

**Fig. 2:**
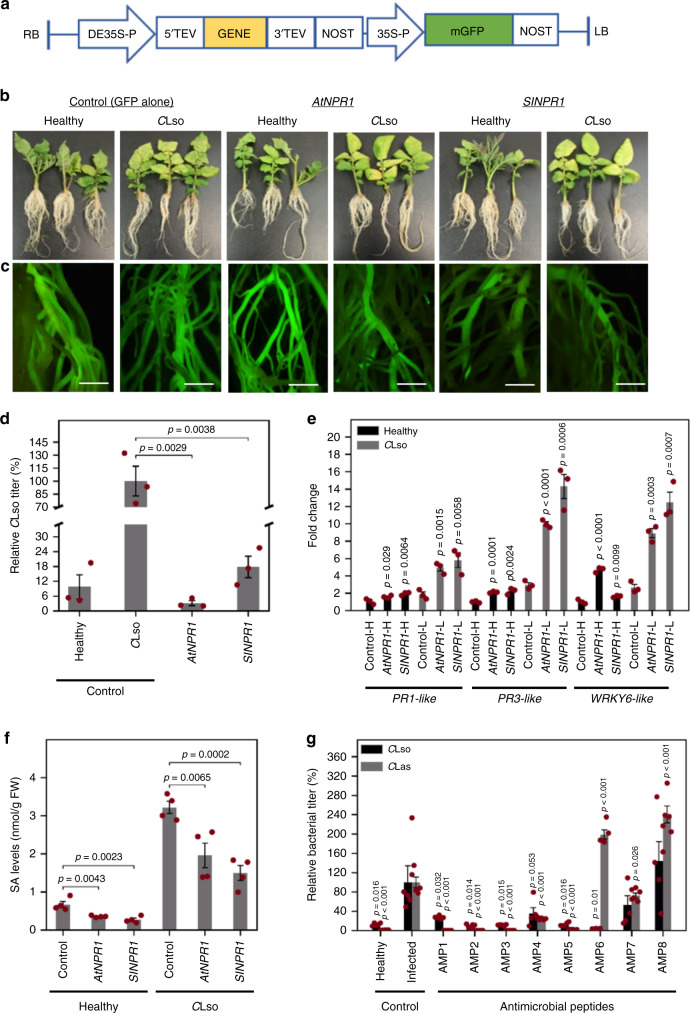
Screening and identification of antimicrobial genes that confer tolerance to *Candidatus* Liberibacter spp. **a** Schematic of a typical transgene overexpression construct with a gene and a GFP marker cassette (included to serve as a visual marker for construct integration in the hairy roots). **b** Representative images of healthy and *C*Lso-potato hairy roots at 30 days post transformation (i.e., 30 dpt) with control (GFP alone) or with putative disease-resistance genes from Arabidopsis (*AtNPR1*) and tomato (*SlNPR1)*. **c** Visualization of the hairy roots under a fluorescence microscope. Scale bars, 1 cm. **d** Quantification of relative *C*Lso titers in transformed hairy roots at 30 dpt, compared to control (GFP alone). Error bars represent ± standard error of mean (*n* = 3). *p* Values were calculated by two-sample *t* test (one-tailed). The experiment was independently repeated two times, and all attempts of replication were successful. **e** Relative expression of *PR1-like* and *PR3-like* and *WRKY6-like* genes in *AtNPR1-* and *SlNPR1-*expressing hairy roots. Error bars represent ± standard error of mean (*n* = 3). *p* Values were calculated by two-sample *t* test (one-tailed) relative to respective control samples. **f** Quantification of salicylic acid (SA) in the transformed hairy roots showed lower SA accumulation in *AtNPR1* and *SlNPR1* overexpressors. Error bars represent ± standard error of mean (*n* = 4). *p* Values were calculated by two-sample *t* test (one-tailed). **g** Evaluation of eight putative antimicrobial peptides in *C*Lso-potato and *C*Las-citrus hairy roots. Relative *C*Lso and *C*Las titers were calculated from five biological replicates. Error bars represent ± standard error of mean (*n* = 5). *p* Values were calculated by two-sample *t* test (one-tailed) relative to infected controls. The experiment was independently repeated two times, and all attempts of replication were successful. Source data underlying Fig. 2d–f are provided as a Source Data file.

Hairy roots are functional plant tissues that mimic the pathogen’s native host root environment. Hence, we leveraged them to conduct functional genetic screening to identify therapeutics that can inhibit *Candidatus* Liberibacter spp. Because *C*Las and *C*Lso are closely related to each other^7–10^, we focused mainly on *C*Lso-hairy potato roots for functional genetic screening due to the faster growth of potato hairy roots compared to citrus hairy roots. First, we evaluated whether gain-of-function genetic studies could be performed using the microbial hairy root system. *Nonexpresser of pathogenesis-related gene 1* (*NPR1*) from *Arabidopsis thaliana* (*AtNPR1* and *AT1G64280*)^45^ is a well-known broad-spectrum immune modulator that is effective against diverse pathogens. NPR1 is a receptor of the defense hormone salicylic acid (SA) and functions as a transcriptional activator of pathogenesis-related (PR) genes in plant defense and systemic acquired resistance (SAR) responses. We set out to evaluate whether any *NPR1* ortholog(s) in *Solanaceae* could confer tolerance to *Candidatus* Liberibacter spp. By phylogenetic analysis (Supplementary Fig. 2) with *AtNPR1* as a reference, we identified an orthologous gene from tomato (*SlNPR1*) and cloned the coding sequences into a binary vector containing a *GFP* reporter gene (Fig. 2a). Then we transformed each *NPR1* construct and an empty vector control containing only *GFP* into unchallenged (healthy) or *C*Lso-infected (*C*Lso) explant tissue for hairy root induction using *R. rhizogenes* (Fig. 2b)^46–49^. Finally, we performed molecular diagnostics on three biological replicates of each type of hairy roots, 30 days after transformation, to confirm the presence of *GFP* and *NPR1* expression in the respective hairy roots (Fig. 2c, Supplementary Fig. 3), followed by a quantitative polymerase chain reaction (qPCR)-based estimation of the *C*Lso titers (Fig. 2d). The heterologous expression of *SlNPR1* significantly (*p* ≤ 0.01) decreased *C*Lso titers (>90%) in the hairy roots compared to the hairy roots transformed with the empty vector (Fig. 2).

To determine the mechanism whereby NPR1 suppressed *Candidatus* Liberibacter spp. in potato, we monitored the changes in the downstream expression of two *PR* genes (*PR-1 like* and *PR-3 like*). Expression of these *PR* genes was significantly higher in both *SlNPR1*- and *AtNPR1*-expressing hairy roots under healthy conditions than in empty-vector-transformed controls (Fig. 2e). These expression levels were further greatly amplified upon *C*Lso infection in both *SlNPR1*- and *AtNPR1*-expressing hairy roots when compared to vector-alone control (Fig. 2e). Expression of a *WRKY* transcription factor (*WRKY6*-like) involved in NPR1-mediated *PR* gene activation^50,51^ was also strongly induced in *SlNPR1*- and *AtNPRI*-expressing hairy roots (Fig. 2e). Compared to healthy hairy roots, *C*Lso-infected hairy roots had significantly higher levels of free SA (Fig. 2f), as determined by liquid chromatography–mass spectrometry (LC–MS/MS). However, despite the activation of downstream defense-related genes, SA levels were significantly lower in *SlNPR1*- and *AtNPR1*-expressing hairy roots than in empty vector controls, under both healthy and *C*Lso-infected conditions (Fig. 2f).

In addition to evaluating plant immune modulators, we tested the efficacy of eight putative broad-spectrum antimicrobial peptides (AMPs) isolated from spinach (*Spinacia oleracea* L.) that show some inhibitory activity against other Gram-positive and Gram-negative bacteria^52–54^. Briefly, we cloned the coding sequences of each peptide into the binary vectors and transformed them into the *C*Lso-potato and *C*Las-citrus explant tissues for hairy root induction and analyzed them in a manner similar to *NPR1* evaluation (Fig. 2b–d). The results of the antimicrobial assays showed that five of the eight AMPs (denoted AMP1–AMP5) significantly (*p* ≤ 0.05 or 0.01) reduced *C*Lso and *C*Las titers (~70–98%) in the hairy roots compared to the controls transformed with the empty vector containing GFP alone (Fig. 2g). Conversely, AMP6 and AMP8 appear to slightly increase *C*Lso and *C*Las titers, possibly by inhibiting other unknown competitive microbes present in the hairy roots.

### Genome editing using microbial hairy roots

**Fig. 3:**
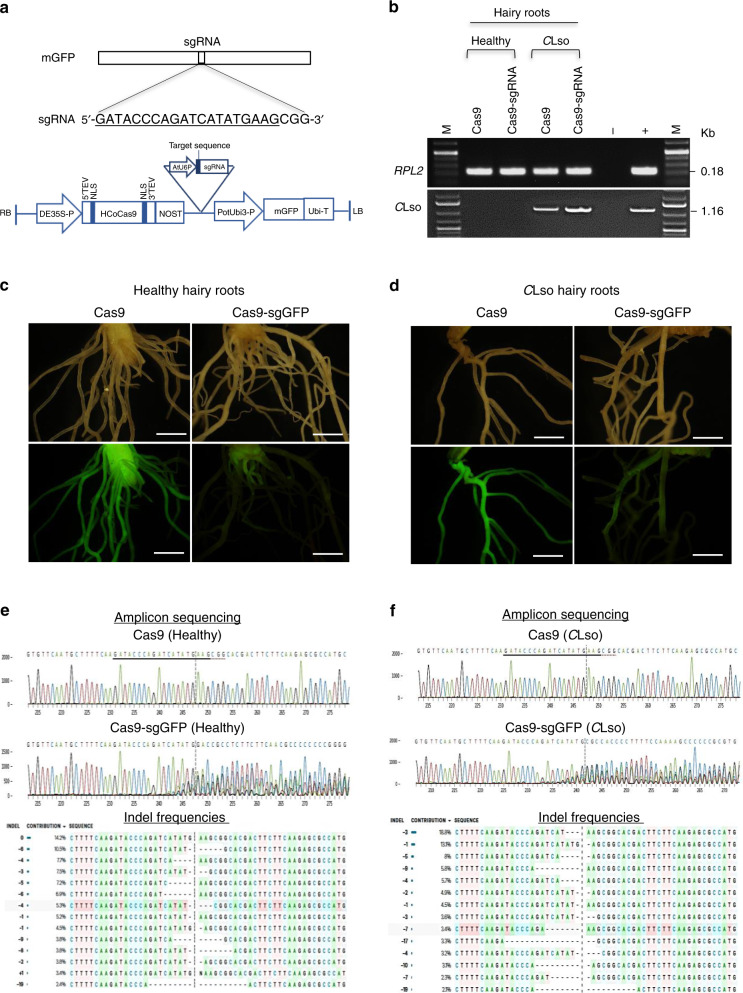
Evaluation of genome editing in microbial hairy roots. **a** Typical CRISPR–Cas9 gene editing construct. The underlined sequence is the target DNA site used in the sgRNA. **b**–**d** Transformation of Cas9 alone (control) and Cas9–sgGFP targeting stably expressed GFP transgene in healthy and *Candidatus* Liberibacter solanacearum (*C*Lso) hairy roots. The loss of GFP fluorescence in Cas9–sgGFP, but not in Cas9 alone, indicates successful editing of the GFP transgene. Scale bars, 1 cm. The experiment was independently repeated two times, and all attempts of replication were successful. **e**, **f** Amplicon sequencing confirmed gene editing in the target site (GFP), as indicated by presence of indels in the Cas9–sgGFP hairy roots, but not in Cas9-alone hairy roots. Frequencies of indels detected were ~86 and 100% in the healthy and *C*Lso hairy roots, respectively. Uncropped raw agarose gel images used to prepare Supplementary Fig. 3b are presented in Supplementary Fig. 11.

Next, we evaluated whether the microbial hairy root system could be used to perform genome-editing studies. For proof-of-concept, we evaluated the editing of a stably integrated *GFP* gene in a genetically engineered potato. To test this hypothesis, we generated constructs containing Cas9 alone (control) or Cas9–sgGFP. We then used these to transform healthy or *C*Lso-containing explant tissues for hairy root induction, as described above (Fig. 3a, b). Fluorescence microscopy imaging of the transformed hairy roots showed a clear loss of GFP fluorescence (from the transgene) (Fig. 3c, d) in hairy roots transformed with Cas9–sgGFP, but not in Cas9 only (control). Amplicon sequencing of the target site confirmed successful editing had occurred in Cas9–sgGFP hairy roots, but not in Cas9 only roots. Frequencies of indels detected were ~86 and 100% in the healthy and *C*Lso hairy roots, respectively (Fig. 3c–f). In addition, we sought to edit an endogenous gene, *NPR3*, in potato. Unlike NPR1, its homologs NPR3 and NPR4 function antagonistically to suppress plant defense in Arabidopsis, and their levels and activities are tightly regulated via complex biochemical interactions in order to maintain defense homeostasis^55–57^. Therefore, it seemed possible that reducing endogenous NPR3 activity in *Solanaceae* could impart plant tolerance for *C*Lso. To test this hypothesis, we identified the potato ortholog of *AtNPR3* (*StNPR3*) and generated constructs containing Cas9 alone (control) or Cas9–sgNPR3. We then used these to transform healthy or *C*Lso-containing explant tissues for hairy root induction, as described above (Supplementary Fig. 4a, b). Amplicon sequencing of the target site confirmed that successful editing had occurred in the Cas9–sgNPR3 hairy roots, but not the Cas9 control hairy roots (Supplementary Fig. 4c), albeit at a lower rate of ~40%, which was not unexpected given the heterozygous autotetraploid nature of potato (2*n* = 4*x* = 48), which means there are four copies of each endogenous gene. Nevertheless, when we estimated the *C*Lso levels of three biological replicates at 30 days post transformation using qPCR, we found that the Cas9–sgNPR3 hairy roots had significantly (*p* ≤ 0.01) lower *C*Lso titers (>90%) than the control hairy roots (Supplementary Fig. 4d). The suppression of *C*Lso titers was strongly associated with induced expression of *NPR1* and several defense marker genes (*WRKY6-like*, *PR-1 like*, and *PR-3 like*) in the Cas9–sgNPR3-edited hairy roots (Supplementary Fig. 4e). Similar activation of plant defenses was observed in *Theobroma cacao* wherein CRISPR editing of ~27% alleles of NPR3 was sufficient to enhance resistance to *Phytophthora tropicalis*^58^. The observed resistance even when partial NPR3 alleles are removed could be due to the systemic nature of the SAR response, whereby a defense signal originated from the NPR3-edited cells is perpetuated to non-edited cells, that ultimately limits the pathogen accumulation. Alternatively, it is possible that native NPR3 activity is affected by gene dosage or haploinsufficiency^59–61^ and/or dominant-negatively inhibited by the truncated/aberrant NPR3 proteins resulting from the edited NPR3 alleles^62^. Regardless of the exact mechanisms of the NPR3 action, these results offer proof-of-concept for conducting genome-editing experiments using the microbial hairy root system and uncover NPR3 as a potential target for CRISPR editing to gain resistance to *Candidatus* Liberibacter spp.

### High throughput in vitro therapeutic screening

**Fig. 4:**
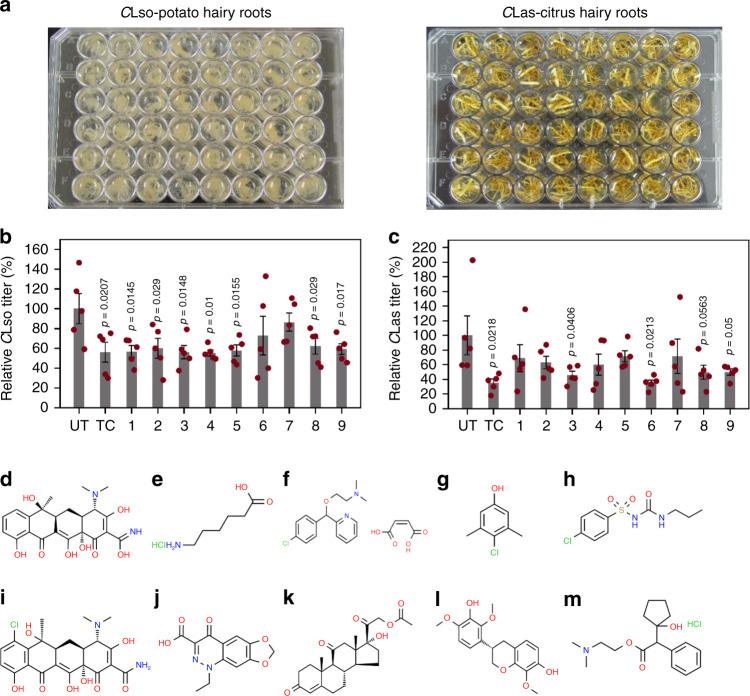
High-throughput screening and identification of small molecules that confer tolerance to *Candidatus* Liberibacter spp. **a**–**c** Multi-well microbial hairy root culture plates were used to conduct high-throughput screening of ~220 compounds (Supplementary Dataset 1). The efficacy data for nine selected hit compounds were re-assayed at 10 and 25 µM, respectively, in **a**, **b** *C*Lso and **a**, **c** *C*Las hairy roots. Untreated (UT) and tetracycline (TC)-treated hairy roots (at 250 and 500 ppm, respectively, for the *C*Lso and *C*Las assays) were used as positive and negative controls. The bacterial titers were estimated by qPCR after 72 h of treatment with each compound and plotted relative to those of untreated samples (set to 100%). Error bars represent ± standard error of mean (*n* = 5). *p* Values were calculated by two-sample *t* test (one-tailed) relative to untreated samples. **d**–**m** Chemical structures of **d** tetracycline and the nine hits: **e** aminocaproic acid (#1), **f** carbinoxamine maleate (#2), **g** chloroxylenol (#3), **h** chlorpropamide (#4), **i** chlortetracycline (#5), **j** cinoxacin (#6), **k** cortisone acetate (#7), **l** duartin (#8), and **m** cyclopentolate hydrochloride (#9). The structures of the different chemical compounds were retrieved from the ChemSpider database (http://www.chemspider.com/) (last accessed on 15 October 2020). Source data underlying Fig. 4b, c are provided as a Source Data file.

The use of *trans*/*cis*-genic-based or genome-editing strategies is a stable, long-term solution to combat diseases; however, it is often a precarious one, owing to the tremendous regulatory burden associated with agricultural use of genetically modified crops, even those produced by CRISPR/Cas. Thus, we also sought to find short- to intermediate-term solutions, and we therefore conducted high-throughput screening of small-molecule inhibitors among a diverse collection of bioactive compounds approved for use in United States and other countries (Microsource Discovery Systems, Inc.)^63,64^. For this, we developed an in vitro multi-well plate assay using the microbial hairy roots, which is scalable from 12- to 48- to 96-well layout. Briefly, we generated microbial hairy roots with *C*Lso and *C*Las, transferred them to a multi-well plate containing growth medium to maintain the hairy roots, and then added small molecules of interest to the assay plates and incubated the hairy roots for 72 h (Fig. 4a). We performed a primary screening with ~220 compounds in *C*Lso hairy roots, using two biological replicates per compound (10 µM) along with untreated or solvent-alone controls (DMSO, 0.1% v/v). By molecular diagnostics, we identified nine molecules that inhibited *C*Lso titers by >50–70% when compared to the untreated controls (Supplementary Dataset 1). We retested these nine molecules using five biological replicates, alongside a reference antibiotic, tetracycline (TC, 250 ppm), that was previously reported to moderately inhibit *C*Las in planta^65^. We also screened the efficacy of the nine compounds (25 µM) and tetracycline (TC, 500 ppm) in *C*Las hairy roots. Seven out of the nine test compounds, as well as tetracycline, consistently showed statistically significant (*p* ≤ 0.05 or 0.01) inhibition of *C*Lso (Fig. 4b). Four compounds showed statistically significant (*p* ≤ 0.05 or 0.01) inhibition of *C*Las, on par with tetracycline (Fig. 4c). Three compounds inhibited both *C*Lso and *C*Las (#3, #8 and #9) (Fig. 4b, c). In general, the active compounds appear to possess diverse structures and bioactive profiles (Fig. 4d–m): three of them were indexed as antibacterial, whereas others were classified as antifungal, antiprotozoal, hemostatic, antihistaminic, antidiabetic, and mydriactic drugs targeting various biological processes. Note that since the retested nine molecules were originally prescreened in *C*Lso-potato hairy roots (Supplementary Data 1), it is very likely that further re-screening of the entire library using *C*Las-citrus hairy roots would identify additional hits.

Besides screening small molecules, we also tested whether the in vitro hairy root bioassay setup could be used for evaluating small peptides (~50 aa). For this, we tested the efficacy of the two AMPs (AMP2 and AMP5; Fig. 2g) by direct vacuum infiltration of the recombinant peptides (5 and 10 µg/ml) into the *C*Las-citrus microbial hairy roots, in a manner similar to the small molecules (Fig. 4a). Molecular diagnostics performed after 72 h of treatment revealed that both AMPs showed statistically significant (*p* ≤ 0.05 or 0.01) dose-dependent inhibition of *C*Las (Supplementary Fig. 5) compared to untreated controls.

**Fig. 5:**
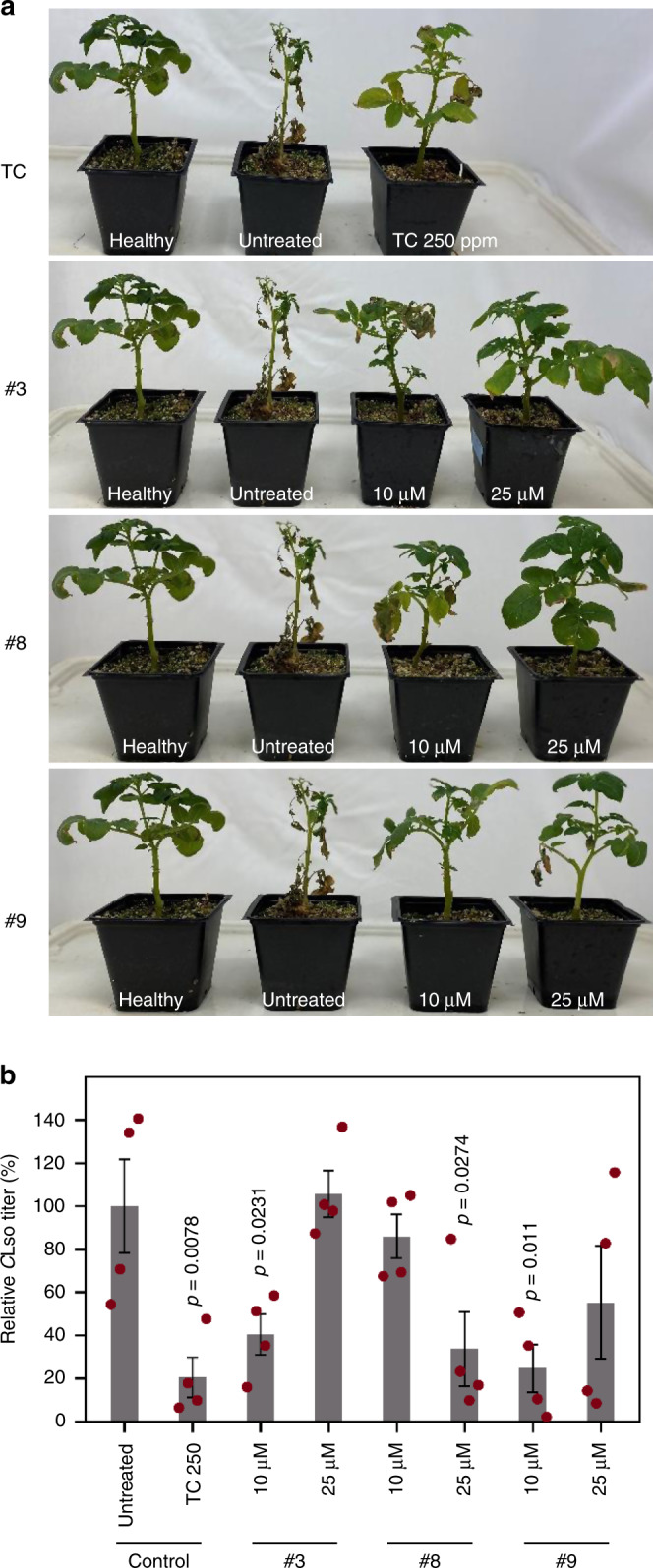
In planta application of small molecules confer tolerance to *Candidatus* Liberibacter spp. in potatoes. **a** Visual symptoms of plants at 28 days post infection (dpi). Tetracycline (TC, 250 ppm) and three selected small molecules (#3, #8, and #9; see Fig. 4) were sprayed on the plants at two concentrations (10 and 25 µM) as indicated. Disease symptoms of chlorosis, necrosis, leaf curling, and wilting were monitored. **b** *C*Lso titers were determined by qPCR at 28 dpi. All titers are plotted relative to those of untreated samples, which were set to 100%. Error bars represent ± standard error of mean (*n* = 4). *p* Values were calculated by two-sample *t* test (one-tailed) relative to untreated samples. Source data underlying Fig. 5b are provided as a Source Data file.

Delivery of small molecules into the plant phloem, especially in hardy perennial citrus trees, is a major challenge. The most practical and common approach is foliar spraying; however, this was shown to be relatively ineffective in citrus as a means to deliver bactericides such as oxytetracycline to inhibit *C*Las^65^. Multiple research groups are evaluating alternative approaches, such as trunk injection and/or nanoparticle-based systems^65,66^. Potato plants are relatively easier to handle and are amenable to foliar spraying techniques. Thus, to determine the in planta efficacy of the three molecules that showed good inhibitory activity against both *C*Lso and *C*Las (#3, #8, and #9) in the hairy root assays (Fig. 4), we used the *C*Lso-potato plant system. First, we repeated efficacy assays in *C*Lso-potato microbial hairy roots, testing a broader range of concentrations (0, 5, 10, 25, and 50 µM) (Supplementary Fig. 6) to establish a general dose-response curve. Based on these results, 10 and 25 µM concentrations were chosen for in planta foliar spraying experiments. Plants were sprayed with each compound twice a week, alongside untreated plants and tetracycline (250 ppm)-treated plants as negative and positive controls, respectively. Disease progression was monitored for 28 days post infection (dpi), by which time untreated plants showed the typical foliar disease symptoms such as chlorosis, necrosis, leaf curling and wilting, and were quite close to dying (Fig. 5a). In contrast, potatoes sprayed with any of the three test molecules showed a lower level of disease symptoms that was also dose-dependent, i.e., plants sprayed with 25 µM showed the least symptoms, followed by those sprayed with 10 µM and untreated plants, and the level of protection was on par with that from tetracycline treatment (Fig. 5a). The attenuated symptoms also correlated with lowered *C*Lso titers in the respective treatment groups compared to untreated controls (Fig. 5b). Together, these experiments demonstrate that the identified compounds can inhibit *Candidatus* Liberibacter spp. in planta, and the efficacy results conform with the results of the hairy root bioassays.

## Discussion

A recent consensus study by the US National Academy of Sciences (NAS) on the status of the citrus greening research and development concluded that there is no single effective therapy to fight the disease^67^. This holds not only for citrus greening but also for other insect-vectored diseases caused by *Candidatus* Liberibacter spp.^1^. The NAS report also suggested deploying a combination of therapies and management practices to combat the disease^54,67–70^. However, a major challenge in finding therapies has been the unculturable nature of *Candidatus* Liberibacter spp., which makes conventional gene- or chemical-therapy evaluation laborious and time-consuming. For instance, evaluating the efficacy of an AMP using either stable transformation or transient viral vectors (e.g., tobacco rattle virus and citrus tristeza virus) followed by disease challenges in mature plants could take from 6 months to several years^71–73^. Similarly, screening of small-molecule inhibitors of active ingredients by foliar spraying, by stem injection, or by grafting using mature plants, is inherently variable due to the challenges associated with delivering the inhibitors to the bacteria that reside unevenly within the plant vasculature^65^. Furthermore, the latter approaches are laborious and time-consuming, and hence not suitable for high-throughput studies^65,68,69^. Researchers have also resorted to using distantly related microbes as surrogates^74–77^. However, these results may or may not be reliable or extrapolatable to the target pathogen, owing to differences in pathosystems and virulence mechanisms (or lack thereof).

In a manner similar to the classical culture strategies for mammalian viruses in host cells, tissues, and embryonated eggs^30–36^, our experiments demonstrate that plant hairy roots can be used for ex vivo propagation of fastidious pathogens like *Candidatus* Liberibacter spp. We hypothesize similar strategies using insect-vector cell lines could be used for propagating *Candidatus* Liberibacter spp. Although this is not an axenic culture system, the microbial hairy root system is highly versatile and enables faster genetic and chemical therapeutic screening, which led to the identification of multiple potential short- and long-term therapies to control *C*Lso and *C*Las. As short-term therapies, we identified eight bioactive compounds that are quite diverse in their chemical structures and modes of action (Fig. 4, Supplementary Dataset 1). Toward long-term solutions, we identified six broad-spectrum AMPs and one positive and one negative plant defense regulator (*SlNPR1* and *StNPR3*, respectively), that inhibit *Candidatus* Liberibacter spp. in plant tissues. All these, either individually or in combinations, could be further deployed in the host plants to develop robust resistance against these phytopathogens.

We also show that the microbial hairy root system can be used to obtain mechanistic insights into gene function. Characterization of *SlNPR1* and *AtNPR1* function in potato hairy roots revealed that *NPR1* confers tolerance to *C*Lso by activating *PR* gene expression, consistent with its known role as a transcriptional co-activator in other plant–microbe interactions. Interestingly, *SlNPR1* and *AtNPR1*-expressing hairy roots accumulate SA to a lesser degree than empty vector controls. There are multiple scenarios that could explain this. First, it is possible that SA accumulation in *NPR1* overexpressors is directly (positively) correlated to the levels of *C*Lso. Second, SA-mediated signaling could be far more potent in the *NPR1* overexpressors than in the controls, and thus less SA might be needed to mediate the defense responses. The second hypothesis posits that in empty vector controls, NPR1 concentration/activity is less than optimal for triggering SA-mediated defenses, and so more SA is produced to compensate for the relatively lower amounts of NPR1. Third, a negative feedback loop in the *NPR1* overexpressors could suppress SA levels to maintain defense homeostasis. Although we did not find any previous reports in which SA levels in *NPR1*-overexpressing plants were determined, studies of Arabidopsis *npr1* mutants and other SA biosynthesis mutants indicate that NPR1 participates in negative feedback regulation of SA biosynthesis^78–82^. Recently, Wang et al.^57^ also showed no concomitant increase in the levels of SA despite activation of two SA-related genes (*PR1* and *PAL1*) in maize roots colonized by *Trichoderma*^83^. Thus, the results here support a model wherein NPR1 mediates *PR* gene activation to inhibit *C*Lso in potato and negatively regulates SA accumulation to prevent toxic build-up and/or to maintain defense homeostasis^78–83^.

A salient feature of the microbial hairy root system is that the efficacy assays are conducted in living (root) tissues that not only mimic the native environment of the pathogen but also encompass features that underpin native plant–microbe interactions, aspects that cannot be replicated in axenic culturing systems. A growing number of studies have indicated the pivotal roles of roots, root–rhizosphere interactions and root–microbiome composition in plant health and disease, including citrus health and HLB progression^84–90^. For instance, in the majority of presymptomatic citrus trees, roots appear to be the first site of *C*Las detection, preceding leaves^86^. *C*Las also is more abundant and more consistently detectable, in roots than in shoots, making root a preferred source tissue in which to perform disease diagnostics tests. Furthermore, roots act as the reservoir for *C*Las between periods of foliar flushing^86^ and to endure the seasonal fluctuations of high temperatures in summer months^84^. Relatively few groups have studied *C*Lso root infections, but the levels and distribution of *C*Lso are greater in roots than shoots, much as for *C*Las^91^. Given the significance of roots to *C*Las and *C*Lso pathology, we posit that the hairy-root-based bioassays described here are more biologically relevant than surrogate pathogen and/or axenic culture systems^74–76^. This is also underscored by reproducible screening results with Arabidopsis *NPR1*, and a broad-spectrum bactericide (tetracycline) (Figs. 2 and 4), which inhibited *Candidatus* Liberibacter spp. in the microbial hairy root bioassays, much as they did in planta (Fig. 5)^65,73^. Moreover, hairy root bioassays circumvent challenges associated with inefficient delivery of compounds into the phloem of whole plants that can result in inconclusive results^65^. For all of these reasons, this approach is very well suited both for primary screening of potential antimicrobial therapies and for narrowing down potential hits.

The hairy-root propagation and antimicrobial screening approaches are relatively straightforward and do not require specialized equipment or apparatus^11–19^. The only prerequisite is that the host plant or variety should be amenable to *R. rhizogenes*-mediated hairy root transformation^92^. Depending on the host plant, the microbial hairy roots can be maintained for ~6 weeks (e.g., *C*Lso-potato hairy roots) or >12 months (e.g., *C*Las-citrus hairy roots) (Fig. 1g, h). In our experience, this is mainly determined by the lifestyle of the host plant (annual vs. perennial), as well as its degree of tolerance for the pathogen. The latter is relevant because the hairy roots are living tissue matrices, and they eventually succumb to pathogen pressure and show vascular degeneration symptoms (Supplementary Fig. 9) in a manner similar to the host plant roots^43,44^. Nevertheless, the hairy root propagation timeframes we have demonstrated are more than sufficient to accomplish various antimicrobial screening experiments.

Another advantage of using the microbial hairy root system is that the screening can be accomplished four to six times faster than with conventional approaches^68,69,71,72^. For instance, small molecules or peptides can be screened using in vitro hairy root assays in ~72 h (Fig. 4), vs. several weeks to months when using conventional in planta approaches (Fig. 5). For evaluating larger proteins, in size range of NPR1, or those forming biochemical complexes as in CRISPR–Cas9, we suggest using the genetic-based hairy root transformation approach (Fig. 2 and Supplementary Fig. 4). Caution must also be exercised to ensure that the small peptides being tested are appropriately folded and/or have the necessary post-translational modifications when produced via synthetic or recombinant techniques. Nevertheless, evaluation of genes/CRISPR targets using genetic constructs still can be accomplished in ~30 days in potatoes and ~90–120 days in citrus after transformation vs. several months to years, respectively, for conventional in planta techniques, thus facilitating faster screening and discovery of therapies.

In conclusion, microbial hairy roots offer a versatile and robust system for the discovery of multiple therapies effective against fastidious pathogens. We suggest that small-molecule inhibitors, AMPs, and genes discovered in this study, either individually or in combination, can be further utilized in planta to control potato ZC and citrus greening diseases.

## Methods

### Plant and insect maintenance and disease challenges

Potato (*Solanum tuberosum* L. var. Atlantic) and tomato (*Solanum lycopersicum* L. var. Lance) plants were propagated in a professional growth mix (Metro-Mix 360, Sun Gro Horticulture, MA) and maintained in a growth chamber at 21–22 °C, with a 14-h/10-h light/dark photoperiod and 50% relative humidity. *Bactericera cockerelli* (Šulc) psyllid colonies that were either *C*Lso positive (haplotype B, the most virulent and prevalent haplotype) or *C*Lso negative were collected in Texas, USA^38^, and maintained in nylon mesh cages (Bugdorm, Taiwan) in the laboratory. The presence and haplotype of *C*Lso in the psyllid colonies were periodically confirmed using PCR, with primers specific to the single sequence repeat (SSR) loci of *C*Lso (SSR-F and SSR-R; Supplementary Table 1)^93^. To generate *C*Lso-infected plants, 1-month-old potato and tomato plants were challenged with ~20 psyllids per plant harboring *C*Lso. The psyllids were released onto the plants in an enclosed organza bag tied to a lower branch with fully expanded leaves and were allowed to feed for 1 week. Subsequently, the bags and psyllids were removed from the plant, and the unchallenged upper branches were sampled periodically using PCR, as described below, to confirm the systemic spread of *C*Lso in the plants, which typically could be detected at around 14 days post challenge.

### Microbial hairy root production and molecular diagnostics

Both ex vivo and in planta approaches were evaluated to generate potato and tomato hairy roots using *C*Lso-infected plants as an inoculum. In the ex vivo approach, branches were excised from either healthy or *C*Lso-infected plants 21 days post challenge and then dipped into a fresh culture of *R. rhizogenes* (American Type Culture Collection strain 15834 or 43056; OD_600_ = 0.5)^47–49^ followed by vacuum infiltration for ~30 min at 30 psi. All explants were subsequently transferred to the inert vermiculite matrix in a humidity dome tray with frequent misting to maintain high humidity. In the in planta approach, *R. rhizogenes* (OD_600_ = 0.5) were directly inoculated into the stems of live plants using a syringe, after which the stems were wrapped with foil to maintain humidity and placed in a growth chamber. To generate the *C*Las-citrus hairy roots, *C*Las-positive branches, confirmed using PCR, were collected from either HLB-graft-infected or naturally infected citrus trees (Rio Red grapefruit [*C*. × *aurantium* L. var. racemosa (Risso & Poit.)] or sour orange [*C*. × *aurantium* L.]) maintained in the research plots of Texas A&M University Kingsville Citrus Center, Weslaco, TX, USA (26°10′00.1″N 97°57′27.7″W). Alternatively, uninfected citrus explants from glasshouse-grown trees could be used for hairy root induction, followed by exposure to the Asian citrus psyllid (*Diaphorina citri*) to transmit *C*Las, although it takes longer to passage *C*Las into hairy roots by the later approach. In either approach, for hairy root induction citrus explants were inoculated with *R. rhizogenes*^46–49^. Briefly, budwood tissues (~0.5 cm diameter, ~5 cm length) with a single node were excised, and the cut ends were immersed in a solution of *R. rhizogenes* (OD_600_ = 0.5), followed by vacuum infiltration for ~30 min at ~30 psi. All explants were subsequently transferred to vermiculite media in a humidity dome tray. The explants were frequently misted with water to maintain high humidity, which is critical for explant viability and hairy root recovery.

Typical hairy root formation in potato and citrus begins approximately 14 and 30 days after transformation, respectively. Upon emergence the authenticity of hairy roots was confirmed using GFP visualization with fluorescence microscopy (Olympus Corporation, Japan). The hairy root transformation efficiency was calculated using the following formula: number of GFP-positive roots/total number of roots × 100. The transformation of the hairy roots was further confirmed using PCR amplification of either the *GFP* or *rolB*/*rolC* genes encoded on the binary and Ri T-DNA plasmids, respectively, which were transformed into the hairy roots. The Solanaceae *RPL2* and citrus *GAPC2* endogenous genes were used as the PCR controls. The presence of *C*Lso in the infected hairy roots were assessed by amplification of *C*Lso 16s rDNA using specific primers (OA2-F/OI2c-R and Lso-F/HLB-R)^37,38^, whereas *C*Las was estimated using specific markers for genes encoding ribosomal protein (*rplA*, A2-F/J5-R) and ribonucleotide reductase β-subunit gene (*nrdB*, RNR-F/RNR-R)^39,40^. Sanger sequencing and BLAST analysis further confirmed the identity of *C*Lso and *C*Las PCR amplicons (Supplementary Fig. 8). The temporal patterns of *C*Lso and *C*Las accumulation in the hairy roots were assessed by determining the titers at various days in propagation. *C*Lso samples were collected at 0, 7, 14, 21, and 28 days, whereas *C*Las samples were collected at 0, 30, 90, 120, and 180 days (Fig. 1g, h). To estimate the copy number, standard curves for *C*Lso and *C*Las were generated and regression equation was used to determine the genome equivalents (GEs), as described below.

### Estimation of *C*Lso and *C*Las copy number using standard curves

Two separate standard curves were established to estimate the copy number and GE of *C*Lso and *C*Las present in the potato and citrus hairy roots, respectively (Supplementary Fig. 7). Briefly, a 76-bp amplicon corresponding to the 16S rDNA of *C*Lso^37^ and an 81-bp amplicon corresponding to the *nrdB* gene of *C*Las^40^ were synthesized and cloned into the pUC57 vector (GenScript, NJ, USA)^37^. This plasmid was used as a template to generate the standard curves. Copy number of these targets was determined from the size of the amplified fragment (76 bp for *C*Lso and 81 bp for *C*Las) and the size of the vector in which the fragment was cloned (2716 + 76 = 2792 bp for *C*Lso and 2716 + 81 = 2797 bp for *C*Las). The molar mass of the plasmid was estimated in Daltons using Eq. 1

1 $${\mathrm{g}}/{\mathrm{mol}} = \left( {{\mathrm{bp}}\,{\mathrm{size}}\,{\mathrm{of}}\,{\mathrm{plasmid}} + {\mathrm{insert}}} \right) \times (330\,{\mathrm{Da}} \times 2\,{\mathrm{nucleotides}}/{\mathrm{bp}}).$$

The weight of a single copy of the plasmid was estimated using Eq. 2

2 $${\mathrm{g}}/{\mathrm{molecule}} = {\mathrm{Weight}}\,{\mathrm{in}}\,{\mathrm{Daltons}}\left( {{\mathrm{g}}/{\mathrm{mol}}} \right)/6.02 \times 10^{23}.$$

Finally, the number of the molecules in a qPCR reaction was determined using the formula: molecules/μL = concentration of plasmid (g/μL)/weight of the one molecule (g/molecule). For the standard curve determination, the plasmid was diluted 10-fold to obtain ~8.17 × 10^9^ to 8.17 × 10^0^ copies and was spiked into 50 ng of DNA extracted from healthy hairy roots for further qPCR analysis. The qPCR analysis was performed as described in the material and methods. The log_10_ value of the copy number was plotted against the threshold cycle (Ct) to establish a standard curve (Supplementary Fig. 7). The standard curve regression equations 3 and 4

3 $$y = - 3.7698x + 39.428,$$

4 $$y = - 3.3197x + 34.822,$$

were used to calculate *C*Lso and *C*Las copy numbers, respectively. Since three copies of the 16S rRNA gene^37^ and five copies of *nrdB* gene^40^ were reported per bacterial genome, to determine the final GE in a test sample, the copy number was divided by total number of copies in each bacterium. The copy number shown in Fig. 1g, h are reported as number of bacterial cells present per 1 ng of hairy root extracted DNA^37^.

### Transmission electron microscopy

Healthy and *C*Lso/*C*Las-infected hairy roots were surface sterilized with 2% bleach, followed by five washes with sterile distilled water. Hairy-root tips (~3 cm) were excised and fixed with 3% (v/v) glutaraldehyde in 0.1 M Sorenson phosphate buffer (pH 7.2) for overnight at 4 °C. Fixed roots were washed in Sorenson phosphate buffer, and post-fixed in 2% osmium tetroxide (w/v) in the same buffer for 4 h at room temperature^94^. All samples were washed in Sorenson phosphate buffer and dehydrated in 10% acetone (v/v), followed by infiltration and embedding in Spurr’s resin over 3 days. Embedded roots were sectioned (100 nm) and mounted on a formvar-coated copper grids (200 mesh), and stained with 2% aqueous uranyl acetate (w/v) and Reynolds lead citrate. Samples were examined under a FEI Morgagni 268 transmission electron microscope (FEI, Netherlands).

### SA quantification

One hundred milligrams of hairy root tissue was mixed with 500 μL phytohormone extraction buffer (1-propanol/water/HCl [2:1:0.002 vol/vol/vol]) containing 500 nM of the internal standard, *d*_6_-SA (Sigma-Aldrich, St. Louis, MO, USA). Zirconia beads were added to the samples, and they were homogenized at 6000 rpm for 1 min at room temperature in a Precellys 24 homogenizer (Bertin Technologies, Montigny-le-Bretonneux, France). The samples were then agitated for 30 min at 4 °C under darkness. Afterwards, 500 μL dichloromethane was added to each sample and the samples were again agitated for 30 min at 4 °C in darkness before being centrifuged at 17,000*g* for 5 min. The lower organic layer of each sample was transferred to a glass vial for evaporation under nitrogen gas. Samples were resuspended in 150 μL methanol, transferred to 1.5-mL microcentrifuge tubes and stored in a −20 °C freezer overnight. The samples were then centrifuged at 17,000*g* for 2 min to pellet any debris, and then 90 µL of the supernatant of each sample was transferred into an autosampler vial for LC–MS/MS analysis. An Ascentis Express C-18 Column (3 cm × 2.1 mm, 2.7 µm) (Sigma-Aldrich, St. Louis, MO, USA) connected to an API 3200 LC–MS/MS (Sciex, Framingham, MA, USA) using electrospray ionization with multiple reaction monitoring was used to detect SA [*m*/*z* = 140.8/96.9 (Q1/Q3)] and *d*_6_-SA [*m*/*z* = 137/92.9 (Q1/Q3)]. The injection volume was 10 μL, and the 500 μL min^−1^ mobile phase consisted of Solution A (0.2% acetic acid in water) and Solution B (0.2% acetic acid in acetonitrile) with a gradient consisting of (time—%B): 0.5–10%, 1.0–20%, 21.0–70%, 24.6–100%, 24.8–10%, 29–stop. SA was quantified by comparison against the isotopically labeled internal standard, d6-SA (St. Louis, MO, USA).

### Antimicrobial efficacy testing using microbial hairy roots

The phylogenetic analysis of Arabidopsis, potato, and tomato *NPR* genes was performed using the Maximum Likelihood statistical method and Jones–Taylor–Thornton substitution model using MEGAX^95^. For the transformation of the antimicrobial genes and CRISPR constructs into the *C*Lso-potato or *C*Las-citrus hairy roots, binary vectors containing either *AtNPR1-*, *SlNPR1-*, or *StNPR3*-CRISPR–Cas9 constructs or the appropriate negative controls (empty vector/GFP alone) were transformed into *R. rhizogenes* and used for the induction of hairy roots using pre-infected explant source material, as described above. About 30 days (for *C*Lso-potato) and 90 days (*C*Las-citrus) after the transformation, the hairy roots were visually screened for GFP under a fluorescence dissection microscope. Three independent biological replicates, comprising randomly selected GFP-positive hairy roots, were collected from the controls and treatments, flash-frozen in liquid nitrogen and stored at −80 °C until use for molecular diagnostics.

High-throughput screening of small molecules was performed using ~220 compounds from the Spectrum bioactives collection (Microsource Discovery Systems, Inc., CT, USA)^63,64^. Primary screening was performed with two biological replicates of each compound (10 µM in aqueous DMSO, 0.1% v/v), along with untreated and solvent-alone negative controls (DMSO, 0.1% v/v) against *C*Lso. Briefly, ~200 mg of the *C*Lso-containing hairy roots were surface sterilized with 2% bleach and then washed five times with sterile distilled water. The hairy roots were transferred into multi-well plates containing Gamborg’s B-5 medium with 1% sucrose. The compounds were then added to the plate, and the medium was vacuum infiltrated at ~20 psi to facilitate the penetration of the molecules into the hairy root matrices. The hairy roots were incubated on a 3D platform rotator (Thermo Fisher Scientific, Waltham, MA, USA) at 60 rpm in the dark at 21 °C for 72 h. The hairy root tissues were sampled after 72 h, flash-frozen in liquid nitrogen and stored at −80 °C in preparation for further processing. The most effective compounds identified in the primary screening were retested at both at 10 and 25 µM, using five biological replicates each, against both *C*Lso and *C*Las, along with tetracycline as a positive control. The structures of the different chemical compounds were retrieved from the ChemSpider database (http://www.chemspider.com/) (last accessed on 15 October 2020). Direct protein infiltration assays were performed in the same manner as for small molecules, but with purified recombinant peptides (Shifa Biomedical Corp., PA, USA) corresponding to AMP2 and AMP5.

### In planta efficacy trials

Three-week-old potato (var. Atlantic) plants were challenged with *C*Lso-containing psyllids. Briefly, five psyllids carrying *C*Lso were restricted to the lowest branch of each plant and contained using an organza drawstring bag tied around the branch. After 1 week, the bags containing psyllids were clipped off the plant to end inoculation and prevent the escape of the newly emerging psyllids and/or nymphs. All the challenged plants were treated with either 0 (untreated), 10 or 25 µM of the selected compounds and tetracycline (250 ppm) twice a week for 4 weeks. To enhance penetration into leaf tissues, all the compounds were mixed with an R-11 nonionic surfactant, which improves the activity and efficacy of the spray application by reducing surface tension and increasing penetration into the plant surface. Four biological replicate plants were included for each treatment. The visual disease symptoms on the plants were monitored weekly, and tissue samples were collected (as described above) at 28 dpi for molecular diagnostics as described below.

### Plasmid design and amplicon sequencing

To obtain the full-length *NPR1* coding sequences, the total RNA was extracted from 3-week-old Arabidopsis (*A. thaliana*; ecotype Col-0) and tomato seedlings and used for cDNA synthesis with SuperScript III Reverse Transcriptase (Thermo Fisher Scientific, Waltham, MA, USA), according to the manufacturer’s instructions. The Arabidopsis *NPR1* (1,782-bp) (*AT1G64280.1*) and tomato *NPR1* (1731 bp) coding sequences (*Solyc07g040690.2.1*) were amplified with Phusion DNA polymerase (New England Biolabs, Ipswich, MA, USA) from the template cDNA using primers containing the *Eco*RI restriction site at the 5ʹ end of the forward primer and a *Bam*HI site at the 3ʹ end of the reverse primer (Supplementary Table 1). The PCR products were then digested with *Eco*RI/*Bam*HI and cloned into a binary vector containing the *GFP* reporter gene (pBIN-mGFP) under the control of a double-enhanced CaMV 35S promoter (DE35S-P) and the 35S terminator (35S-T). All the nucleotide sequences were confirmed using Sanger DNA sequencing before being transformed into *R. rhizogenes* to generate hairy roots.

For the CRISPR experiments, multiple single guide RNA (sgRNA) targets were designed for the *GFP* and *NPR3* gene sequences (*PGSC0003DMG401000923*) using the CRISPR-P design toolset^96^. The sequences of the top sgRNA hits are provided in Fig. 3a and Supplementary Fig. 4a. The respective sgRNA targets were produced as synthetic oligos (Integrated DNA Technologies, Inc., IA, USA) with the appropriate overhang sequences. Each pair of oligonucleotides was annealed and phosphorylated. The pChimera vector containing the Arabidopsis U6-26 promoter^97^ was linearized using *Bbs*I, and the spacer was ligated between the two *Bbs*I sites using the annealed oligonucleotides. The customized RNA chimera was then subcloned into pBIN-HcoCas9:mGFP^98^ to obtain a functional Cas9 expression construct for targeted mutagenesis (Fig. 3a). The Cas9 vector carrying *sgNPR3* or the *sgGFP* spacer sequence and Cas9 were independently transferred into *R. rhizogenes* for the generation of the hairy roots.

To detect indels in the transgenic hairy roots, a 500-bp target region in *NPR3* or *GFP* was amplified using PCR with gene-specific primers (Supplementary Table 1) and Phusion Polymerase (New England Biolabs). The PCR amplicons were cleaned using DNA PCR clean-up kits (Zymo Research, Irvine, CA, USA) and sequenced using Sanger sequencing (Eton Bioscience, Inc., San Diego, CA, USA). The percentage of indels in the transgenic hairy roots was analyzed using the Inference of CRISPR Edits tool^99^.

### Molecular diagnostics

To confirm that the transgene was expressed in the transformed hairy roots, total RNA was isolated from the transformed materials using the Direct-zol RNA MiniPrep Plus kit (Zymo Research) according to manufacturer’s instructions and then subjected to reverse transcription PCR. First-strand cDNAs were synthesized from 2.0 μg total RNA using the Superscript III First-Strand Synthesis System (Thermo Fisher Scientific), following the manufacturer’s instructions. Subsequently, PCR analysis was performed using a ProFLex PCR System (Thermo Fisher Scientific) to validate gene expression in the hairy roots. The tomato and potato *RIBOSOMAL PROTEIN L2* (*RPL2*) genes (accessions *Solyc10g006580.2* and *PGSC0003DMG400025015*, respectively) and citrus *GLYCERALDEHYDE-3-PHOSPHATE DEHYDROGENASE C2* (*GAPC2*, *XM_006483974.2*) were used as endogenous reference genes for the normalization and estimation of the relative expression levels. The primer pairs used are listed in Supplementary Table 1 and targeted the following: the 5′ and 3′ untranslated regions (UTRs) of Tomato Etch Virus (TEV) flanking the *NPR1* genes (TEV-F and TEV-R) (Fig. 2a), tomato *RPL2* (RPL2-F and RPL2-R)^100^, potato *RPL2* (StRPL2-F and StRPL2-R), and citrus *GAPC2* (GAPC2-F and GAPC2-R)^101^.

To determine the effects of small molecules, transgenes and the CRISPR–Cas9 constructs on the *C*Lso and *C*Las titers within the hairy roots, qPCR analysis was performed using DNA isolated from the various samples. DNA was isolated from the citrus hairy roots from ~200 mg of tissue homogenized using a Precellys 24 homogenizer (MO BIO Laboratories, Carlsbad, CA, USA)^102^. The quality and quantity of DNA were determined using a NanoDrop 1000 Spectrophotometer (Thermo Fisher Scientific) and agarose gel electrophoresis. Approximately, 50 ng total DNA was used for the qPCR analysis, which was performed on a CFX384™ Real-Time PCR Detection System (Bio-Rad Laboratories) using iTaq™ universal SYBR^®^ Green supermix (Bio-Rad Laboratories), following the manufacturer’s instructions. The *C*Lso 16S rDNA-specific primers (OA2-F/OI2c-R and Lso-F/HLB-R) were used to detect *C*Lso^37,38^, whereas primers for the *C*Las genes encoding ribosomal protein (*rplA*, A2-F/J5-R) and ribonucleotide reductase β-subunit (*nrdB*, RNR-F/RNR-R) were used to detect *C*Las^39,40^ (Supplementary Table 1). A dissociation or melting curve analysis was performed to confirm the lack of nonspecific amplification and primer-dimers. For the antimicrobial treatments, the relative *C*Lso titers were estimated using the 2^−ΔΔ^Ct method^103^. The *C*Lso Ct values were normalized to *RPL2* to correct for concentration differences in the DNA templates among the samples and were plotted relative to the level in the control (DMSO-treated) *C*Lso hairy root samples, which was set to 100%. Student’s *t* test was used to determine statistically significant (*p* ≤ 0.05 or 0.01) differences among the controls and treatments.

### Data analysis and statistics

All the data analysis, statistics and graphs were displayed as described in the figure legends using Microsoft Excel software (version 2009) and Python bioinfokit package (https://github.com/mandadi-lab/bioinfokit).

### Reporting summary

Further information on research design is available in the Nature Research Reporting Summary linked to this article.

## Supplementary information

Supplementary Information

Peer Review File

Reporting Summary

Description of Additional Supplementary Files

Supplementary Data1

## Data Availability

The data supporting the findings of this study are available in the Supplementary Information Files. A reporting summary for this article is available as a Supplementary Information file. The datasets and/or plant materials generated and analyzed during the current study are available from the corresponding author upon request. Source data are provided with this paper.

## Source data

Source Data
