# Supplementary material for: Plant hairy roots enable high throughput identification of antimicrobials against Candidatus Liberibacter spp
Source: Nat Commun. 2020 Nov 16;11:5802. doi: 10.1038/s41467-020-19631-x (PMC7669877; doi:10.1038/s41467-020-19631-x)
Supplement: Supplementary file 1 — Supplementary Information [file 41467_2020_19631_MOESM1_ESM.pdf]

Plant hairy roots enable high throughput identification of antimicrobials against *Candidatus* Liberibacter spp.

Irigoyen *et al.*

Supplementary Table 1. Primers used in this study.

Primer name	Sequence (5′-3′)	Reference
AtNPR1-F	CCGAATTCGTATGGACACCACCATTGATGGA	Current study
AtNPR1-R	CCGGATCCGTTCACCGACGACGATGAGAGA	Current study
SlNPR1-F	CCGAATTCGTATGGATAGTAGAACTGCTTTTTCGG	Current study
SlNPR1-R	CCGGATCCGTCTATTTCCTAAATGGGAGATTATTG	Current study
*St*NPR3-G1-F	ATTGGTCGTTGAGGGGATTGATGT	Current study
*St*NPR3-G1-R	AAACACATCAATCCCCTCAACGAC	Current study
StNPR3-F	TTTGAACAATGGAGAGCGGC	Current study
StNPR3-R	TAATTAATGGCAGGGCGGCA	Current study
TEV5′-F	ACGAATCTCAAGCAATCAAGC	Current study
TEV3′-R	CTCACTTTGTCGTTCGCCTA	Current study
SSR-F	TTATTTTGAGATGGTTTGTTAAATG	[1]
SSR-R	TATTATCATTCTATTGCCTATTTCG	[1]
SlRPL2-F	GAGGGCGTACTGAGAAACCA	[2]
SlRPL2-R	CTTTTGTCCAGGAGGTGCAT	[2]
StRPL2-F	GTGGAGGACGAACTGAGAAA	Current study
StRPL2-R	AGTCCTCCTTGCAGCAATAA	Current study
CsiGAPC2-F	GAGGAGATCCCATGGGCAAA	[3]
CsiGAPC2-R	AAGAGGAGCTAGGCAGTTGG	[3]
OA2-F	GCGCTTATTTTTAATAGGAGCGGCA	[4]
OI2c-R	GCCTCGCGACTTCGCAACCCAT	[4]
Lso-F (qPCR)	CGAGCGCTTATTTTTAATAGGAGC	[5]
HLB-R (qPCR)	GCGTTATCCCGTAGAAAAAGGTAG	[5]
A2-F	TATAAAGGTTGACCTTTCGAGTTT	[6]
J5-R	ACAAAAGCAGAAATAGCACGAACAA	[6]
RNR-F (qPCR)	CATGCTCCATGAAGCTACCC	[7]
RNR-R (qPCR)	GGAGCATTTAACCCCACGAA	[7]
RolB-F	GCTCTTGCAGTGCTAGAT	[8]
RolB-R	GAAGGTGCAAGCTACCTCC	[8]
RolC-F	CTCCTGACATCAAACTCG	[8]
RolC-R	TGCTTCGAGTTATGGGTA	[8]
mGFP-F	CTCTTTCTCATCTTTTCACTTCTCC	Current study
mGFP-R	GGACAGGTAATGGTTGTCTGGT	Current study
StPR1-F (qPCR)	CTGGTGCTGTGAAGATGTGG	Current study
StPR1-R (qPCR)	ACAACCAAGACGTACCGAGT	Current study
StPR3-F (qPCR)	GCAAATTCGGCTGGTGTGGTA	Current study
StPR3-R (qPCR)	CTGGAGAACCGCCAGGACAC	Current study
StWRKY1-F (qPCR)	GGCGAAAGGCAATCCATGTC	Current study
StWRKY1-R (qPCR)	TTGTCCGATCCTCAGCACAC	Current study
StNPR1-F (qPCR)	GCCGATGATCTGCCCATGAA	Current study
StNPR1-R (qPCR)	GGTTCTTTGGCCTTGATTGGT	Current study

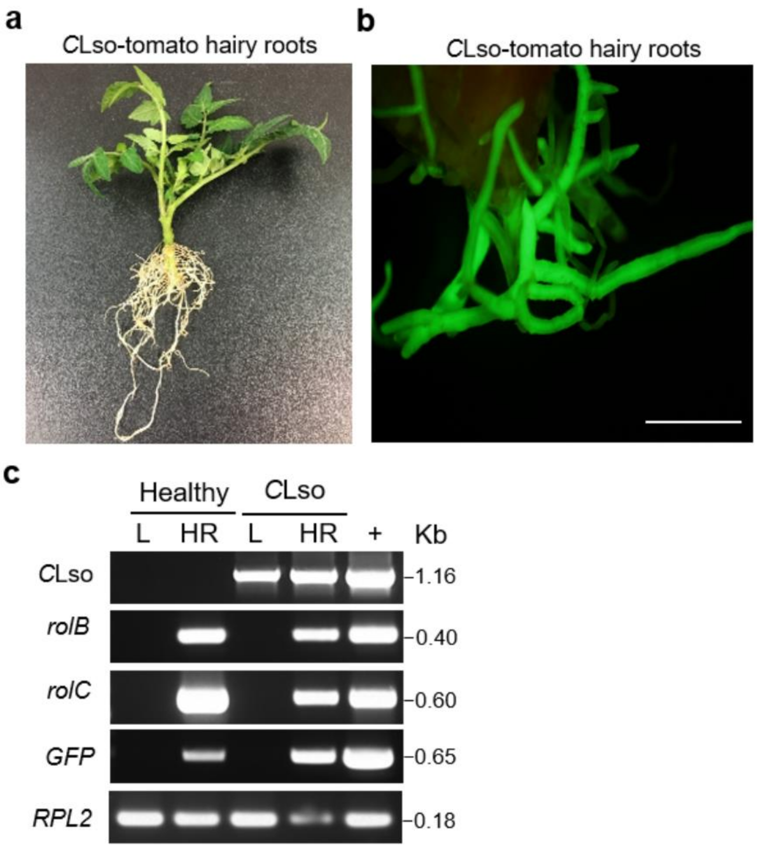

Supplementary Fig. 1. ***Candidatus*** **Liberibacter solanacearum in tomato hairy roots. a**, Hairy root production using *Candidatus* Liberibacter solanacearum (*C*Lso)-infected tomato explants. **b**, Visual confirmation of GFP expression in hairy roots by fluorescence microscopy. Scale bar, 1 cm. **c**, Detection of *C*Lso in hairy roots by PCR amplification of diagnostic markers specific to *C*Lso (16S rDNA). *GFP*, *rolB* and *rolC*, encoded on the Ti and Ri plasmids respectively, and co-transformed into the hairy roots, were used as additional markers for hairy root authenticity; *RPL2* is an endogenous tomato gene, used as genomic DNA control for PCR. 'L' and 'HR' indicate leaf and hairy root sample, respectively. '+' indicates a positive control used for the respective PCR amplifications. The experiment was independently repeated two times, and all attempts of replication were successful. Uncropped raw agarose gel images used to prepare Supplementary Fig. 1c are presented in Supplementary Fig. 11.

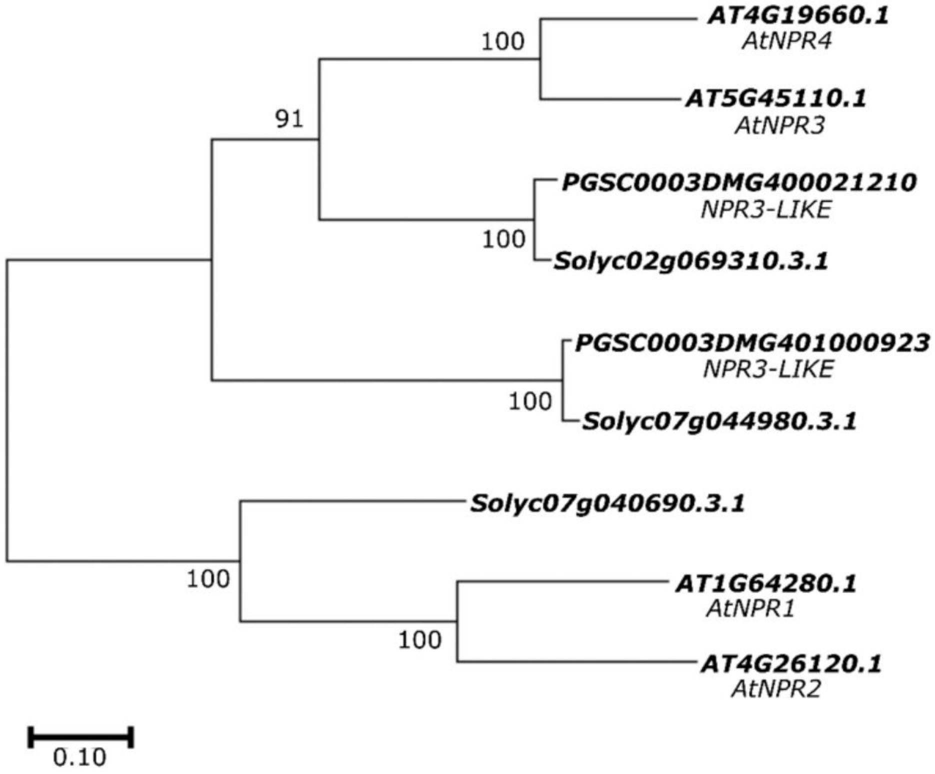

Supplementary Fig. 2. Phylogenetic analysis of the Arabidopsis, tomato, and potato *Non-expressor of Pathogenesis Related* (*NPR*) gene families. Phylogenetic tree inferred by the maximum-likelihood method using amino acid sequences of NPR-related members from Arabidopsis, potato, and tomato. The tree is drawn to scale, with branch lengths measuring the number of substitutions per site. Bootstrap values are indicated by the numbers at the branch points.

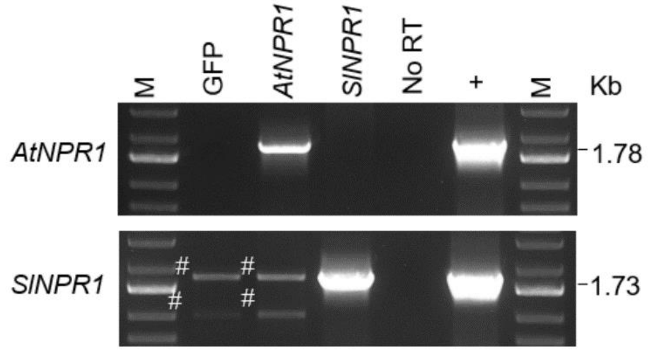

Supplementary Fig. 3. Confirmation of *NPR1* expression in *C*Lso-potato hairy roots. Hashtags (#) indicate the amplification of a putative endogenous potato *NPR1*, which was amplified in both control (GFP) and *AtNPR1*-transformed potato hairy roots. '+' indicates a positive control used for the respective PCR amplifications. 'M' indicates DNA molecular marker. Uncropped raw agarose gel images used to prepare Supplementary Fig. 3 are presented in Supplementary Fig. 11.

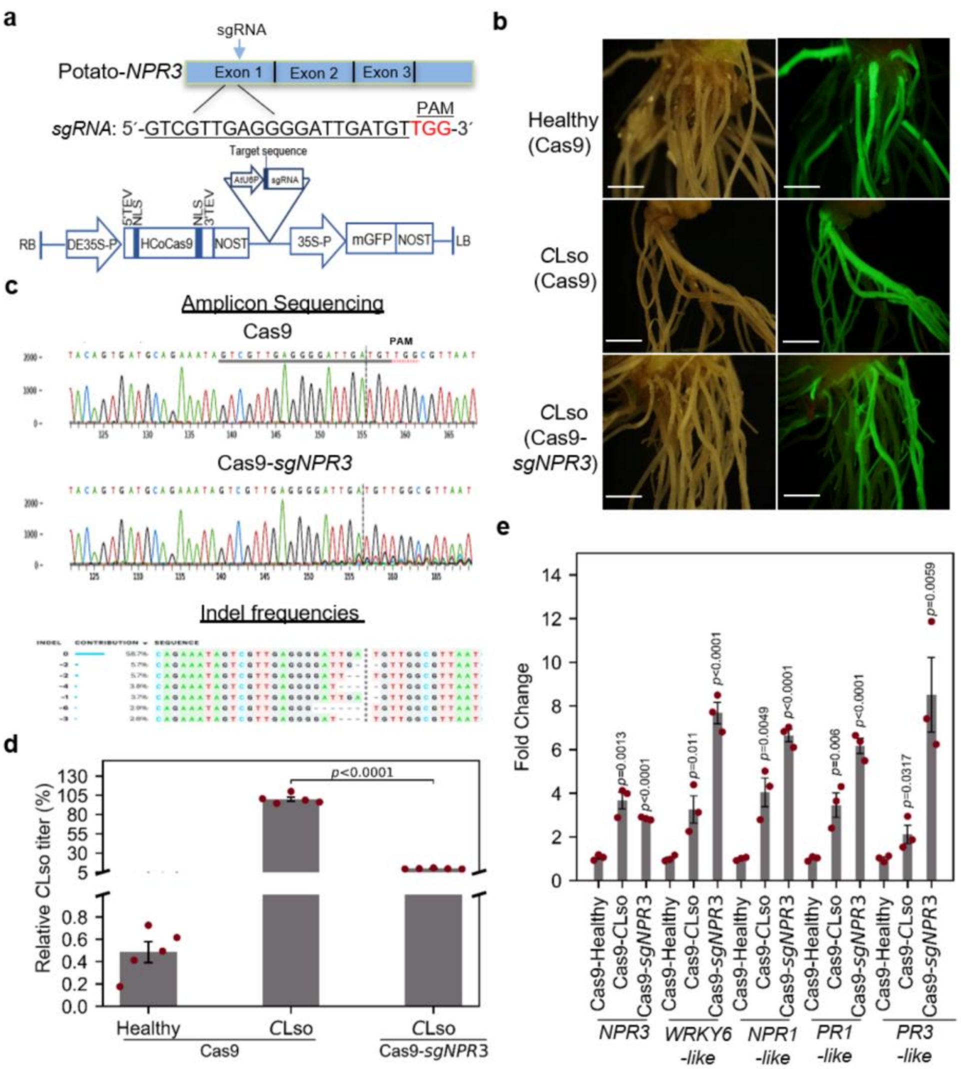

Supplementary Fig. 4. Genome editing of *StNPR3* in potato hairy roots confers tolerance to *Candidatus* Liberibacter spp. (**a**) Schematic of CRISPR-Cas9 construct with the potato *NPR3* sgRNA and GFP marker cassette (included to serve as a visual marker for construct integration in the hairy roots). (**b**) Transformation with Cas9 alone (control) and Cas9-sgNPR3 targeting potato *NPR3* in healthy and '*Candidatus* Liberibacter solanacearum' (*C*Lso)-infected hairy roots. Scale bar, 1 cm. (**c**) Amplicon sequencing confirming gene editing in the target DNA (*StNPR3*), as indicated by presence of indels in the Cas9-sgNPR3 hairy roots, but not in Cas9 alone. (**d**) Quantification of *C*Lso in edited hairy roots. Error bars represent ± standard error of mean (n=5). *p*-values were calculated by two-sample *t*-test (one-tailed) relative to Cas9-*C*Lso samples. The experiment was independently repeated two times, and all attempts of replication were successful. (**e**) Relative expression of the genes *WRKY6-like, NPR1-like, PR1-like* and *PR3-like* in *StNPR3*-edited hairy roots. Error bars represent ± standard error of mean (n=3). *p*-values were calculated by two-sample *t*-test (one-tailed) relative to Cas9-Healthy samples. Source data underlying Supplementary Figure 4d and 4e are provided as a Source Data file.

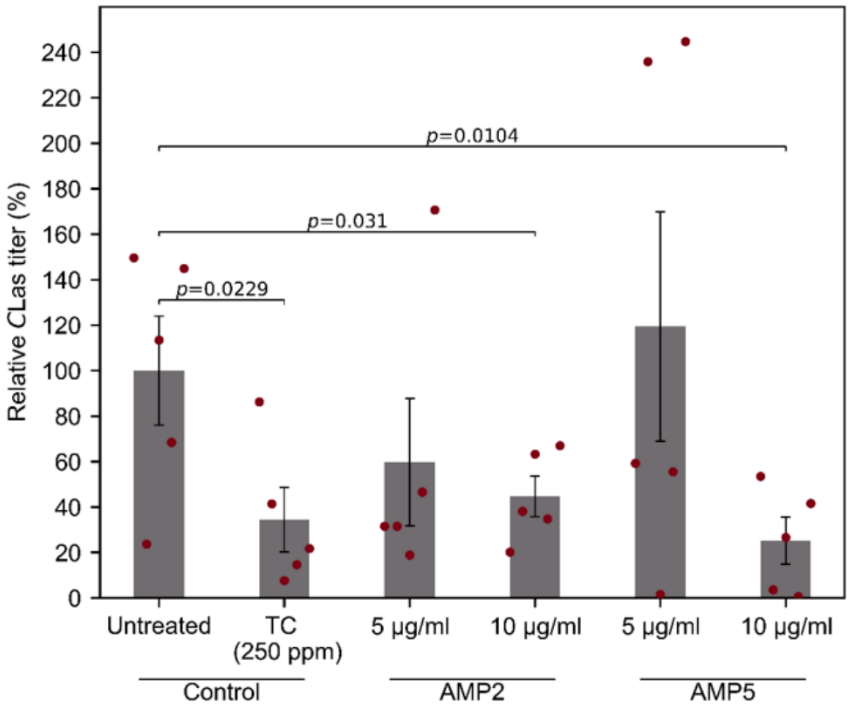

Supplementary Fig. 5. Efficacy testing of antimicrobial peptides AMP2 and AMP5 by direct infiltration in microbial hairy roots. Relative *C*Las titers were calculated from five biological replicates. Untreated and tetracycline-treated (TC, 250 ppm) hairy roots were used as negative and positive controls. Error bars represent ± standard error of mean (n=5). *p*-values were calculated by two-sample *t*-test (one-tailed) relative to untreated samples. Source data are provided as a Source Data file.

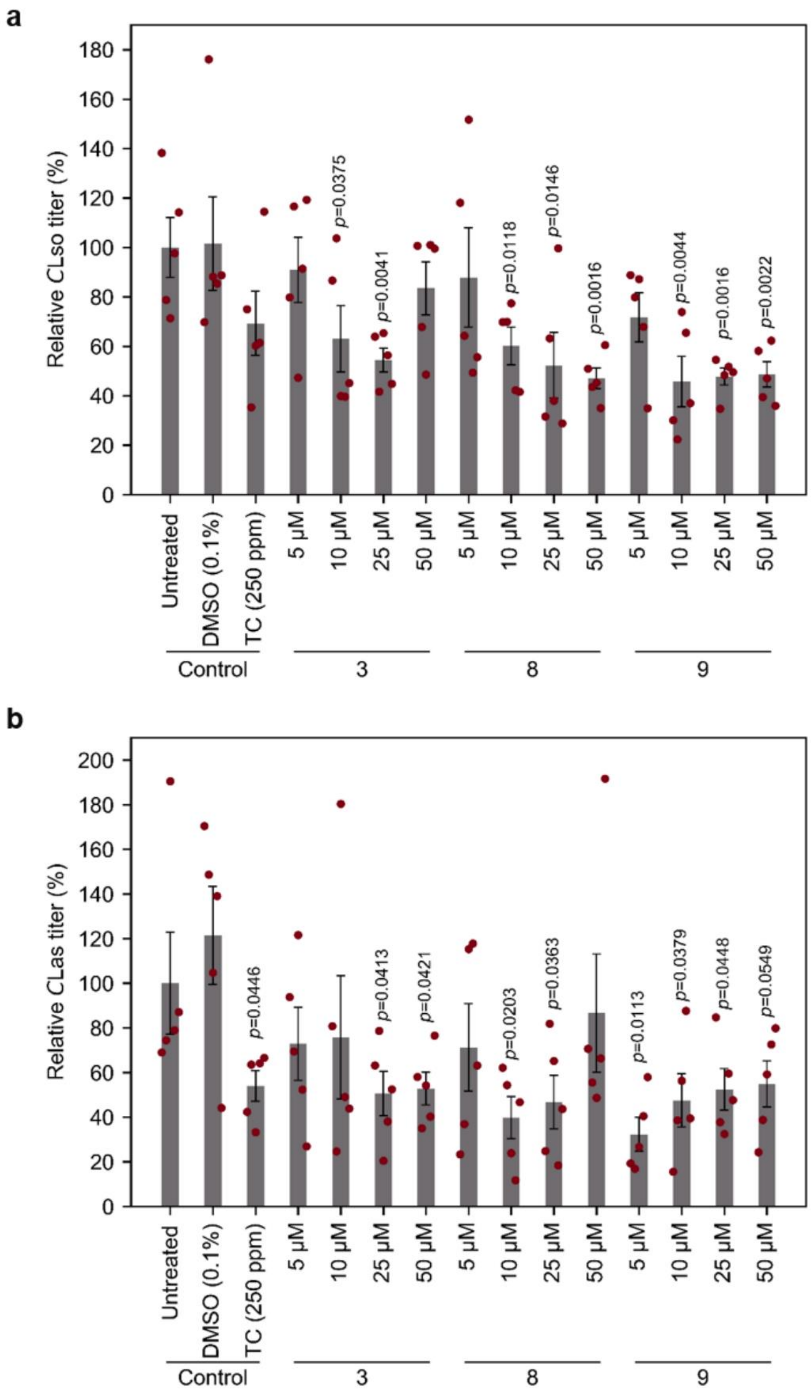

Supplementary Fig. 6. Dose-response inhibition assays of three selected compounds in *C*Lso-potato and *C*Las-citrus hairy roots. Dose-response inhibition assays of three selected compounds in *C*Lso-potato and *C*Las-citrus hairy roots**.** Relative *C*Las (**a**) and *C*Lso (**b**) titers were calculated from five biological replicates. Untreated, DMSO (0.1%) and tetracycline (TC, 250 ppm) were used as negative and positive controls. Error bars represent ± standard error of mean (n=5). *p* values were calculated by two-sample *t*-test (one-tailed) relative to untreated samples. Source data are provided as a Source Data file.

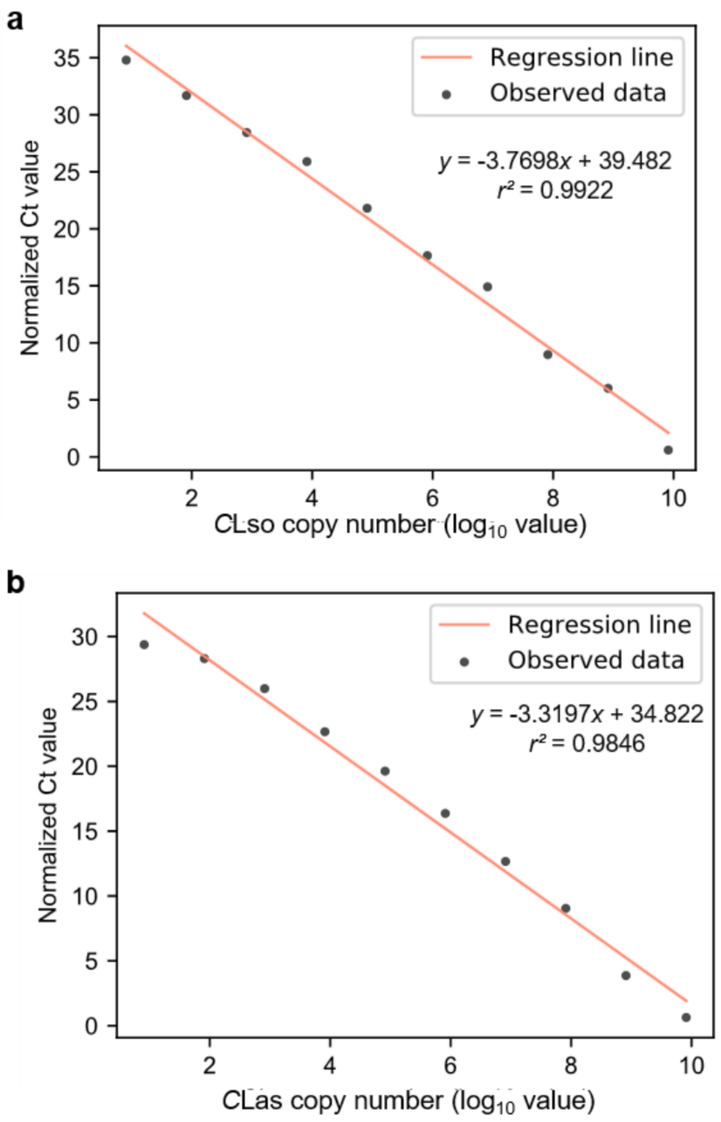

Supplementary Fig. 7. Standard curves and regression analysis to establish relationship between Ct and *C*Lso or *C*Las copy number. Two separate standards curves were established for quantification of *C*Lso copy number in potato (**a**) and *C*Las copy number in citrus (**b**). A plasmid containing the *C*Lso and *C*Las target amplicons corresponding to the 16S rRNA gene and *nrdB* gene was synthesized and spiked into 50 ng of genomic DNA extracted from the respective hairy root tissues in 10-fold dilutions yielding 8.17×10^9 to 8.17×10^0 copies in each sample. Samples were subjected to qPCR to determine the target Ct value, and the data presented are the average normalized Ct of three replicates. The estimated correlation coefficient (r^2) was 0.99 and 0.98 for *C*Lso and *C*Las, respectively suggesting good linear range of the data. The above listed regression equations were used to calculate *C*Lso and *C*Las copy number and genome equivalents in other samples as described in Methods. Source data are provided as a Source Data file.

a

>*C*Las (Primer specific to *C*Las ribosomal protein L1, *rplA* gene)

```
CCNTGACTACTCTTGTTTAGTTGTTTTTTGTGTGGATTCCTTTTTCGCTATCGGATCGCTTCTTTTTTGTAAGGGA
TGCGTTAGGATTTTTGTTCTTCTTCGAAATCAAGATATGAAAATATTTTCTTGGTATAGATATAGGAAAAGGAATG
GGTATATTTGTCATCTGGAGATGAAAGTTGAATAGACAAGGAAAGAGCGTAGAAATTTCTGAATTAAGTAAGATTT
TTTCTTCTTCTGGATCAATTGTTGTTGCACATTATAAGGGAATTAGTGTTGCGCAAATTAAAGATCTTCGGAAAAA
GATGCGGGAAGCTGGTGGAGGTGTAAAAGTTGCCAAAAATCGTCTCGTCAAGATTGCTATCCGTGATACTAGTATT
AGAGGAATATCTGATCTTTTCGTTGGGCAGTCTCTAATTGTCTATTCGGATAGTCCTGTTATTGCTCCTAAAATTT
CGGTTAGCTTTTCAAATGACAATAATGAATTTAGAGTTCTTGGTGGGGTTGTAGAGAAGGGCGTCCTTAATCAAGA
TTCTATCAAGCAAATTGCTTCTTTACCCGATCTTGAGGGTATTCGAGCTGGTATCATAAGTGCTATCCAATCTAAT
GCGACTACATTGGTTAGACTTCTTGGTACGCGTCAGACTCAAATTGTTCTTGCGGTTCCCGCACCACCTTTGCCTC
AGCTCAGAAGGTGACGATTAAATAATCCTATACTTACG
```

Sequences producing significant alignments — Download — Manage Columns — Show 100

select all *100 sequences selected* — GenBank — Graphics — Distance tree of results

Description	Max Score	Total Score	Query Cover	E value	Per. Ident	Accession
Candidatus Liberibacter asiaticus strain A4, complete genome	1175	1175	90%	0.0	99.08%	CP010804.2
Candidatus Liberibacter asiaticus isolate Gondar_GNDGJ ribosomal protein L1 (rplA) and ribosomal protein L10 (rplJ) genes, partial cds	1175	1175	90%	0.0	99.08%	MK542517.1
Candidatus Liberibacter asiaticus isolate Dangila_TE_72 ribosomal protein L1 (rplA) and ribosomal protein L10 (rplJ) genes, partial cds	1175	1175	90%	0.0	99.08%	MH809485.1
Candidatus Liberibacter asiaticus isolate K5 50S ribosomal subunit protein L1 (rplA) and 50S ribosomal subunit protein L10 (rplJ) genes, partia	1175	1175	90%	0.0	99.08%	MF694639.1
Candidatus Liberibacter asiaticus isolate MG 50S ribosomal subunit protein L1 (rplA) and 50S ribosomal subunit protein L10 (rplJ) genes, parti	1175	1175	90%	0.0	99.08%	MF769717.1
Candidatus Liberibacter asiaticus isolate B1 50S ribosomal subunit protein L1 (rplA) and 50S ribosomal subunit protein L10 genes, partial cds	1175	1175	90%	0.0	99.08%	MF769716.1
Candidatus Liberibacter asiaticus isolate AK4 50S ribosomal subunit protein L1 (rplA) and 50S ribosomal subunit protein L10 (rplJ) genes, part	1175	1175	90%	0.0	99.08%	MF769715.1
Candidatus Liberibacter asiaticus isolate AK9 50S ribosomal subunit protein L1 (rplA) and 50S ribosomal subunit protein L10 (rplJ) genes, part	1175	1175	90%	0.0	99.08%	MF769714.1
Candidatus Liberibacter asiaticus isolate SK16 50S ribosomal subunit protein L1 (rplA) and 50S ribosomal subunit protein L10 genes, partial c	1175	1175	90%	0.0	99.08%	MF769713.1

b

>*C*Lso (Primer specific to *C*Lso 16S ribosomal rRNA gene)

```
NNGCCCTGTAGCCGTGTGTAGCCCAGCCCATAAGGGCCATGAGGACTTGACGTCATCCCCACCTTCCTCCGGATTAT
CACCGGCAGTCCCTATAAAGTACCCAACATCTAGATAAAATCTAAACTTGATGGCAACTAGAGGTAGGGGTTGCGCT
CGTTGCGGGACTTAACCCAACATCTCACGACACGAGCTGACGACAGCCATGCAGCACCTGTATAAAGGTCTCCGAAA
AGAAAATACCATCTCTGATATCGTCCTCTATATGTCAAGGGCTGGTAAGGTTCTGCGCGTTGCATCGAATTAAACCA
CATGCTCCACCGCTTGTGCGGGCCCCCGTCAATTCCTTTGAGTTTTAATCTTGCGACCGTACTCCCCAGGCGGAGTG
CTTAATGCGTTAGCTGCGCCACTGAATGGTAAACCACCCAACAGCTAGCCCTCATCGTTTACAGAATGGACAACAAG
GGTATCTAATCCTGTTTGCTCACCATGAATTTCATAATCTTCTTTCGAAATAAAGCCAAAGCACTGCATACTCCAAT
GGTGTTACCAACAATACCTAAGAATATCTCACAACACATTCAGTATCAACTAAAA
```

Sequences producing significant alignments — Download — Manage Columns — Show 100

select all *100 sequences selected* — GenBank — Graphics — Distance tree of results

Description	Max Score	Total Score	Query Cover	E value	Per. Ident	Accession
Candidatus Liberibacter solanacearum clone M8-C-Pi 16S ribosomal RNA gene, partial sequence	850	850	80%	0.0	98.55%	MT036060.1
Candidatus Liberibacter solanacearum clone M7-T-Pi 16S ribosomal RNA gene, partial sequence	850	850	80%	0.0	98.55%	MT036059.1
Candidatus Liberibacter solanacearum clone M6-T-Pi 16S ribosomal RNA gene, partial sequence	850	850	80%	0.0	98.55%	MT036058.1
Candidatus Liberibacter solanacearum clone M5-T-Im 16S ribosomal RNA gene, partial sequence	850	850	80%	0.0	98.55%	MN396641.1
Candidatus Liberibacter solanacearum clone M4-T-Im 16S ribosomal RNA gene, partial sequence	850	850	80%	0.0	98.55%	MN396640.1
Candidatus Liberibacter solanacearum clone M3-T-Pi 16S ribosomal RNA gene, partial sequence	850	850	80%	0.0	98.55%	MN396639.1
Candidatus Liberibacter solanacearum isolate AB171 16S ribosomal RNA gene, partial sequence	850	850	80%	0.0	98.55%	MH843709.1
Candidatus Liberibacter solanacearum isolate 1 16S ribosomal RNA gene, partial sequence	850	850	80%	0.0	98.55%	KX185608.1
Candidatus Liberibacter solanacearum isolate YARL1 16S ribosomal RNA gene, partial sequence	850	850	80%	0.0	98.55%	KU588194.1
Candidatus Liberibacter solanacearum isolate 2 16S ribosomal RNA gene, partial sequence	850	850	80%	0.0	98.55%	KX197200.1

Supplementary Fig. 8. Sanger sequencing and BLAST analysis confirms the identities of (a) *C*Las and (b) *C*Lso in the citrus and potato hairy roots, respectively. The respective PCR amplicons were sequenced, and BLAST analysis was performed to determine the identity of the PCR product. The top ten hits shown are hits to corresponding *C*Las and *C*Lso gene sequences.

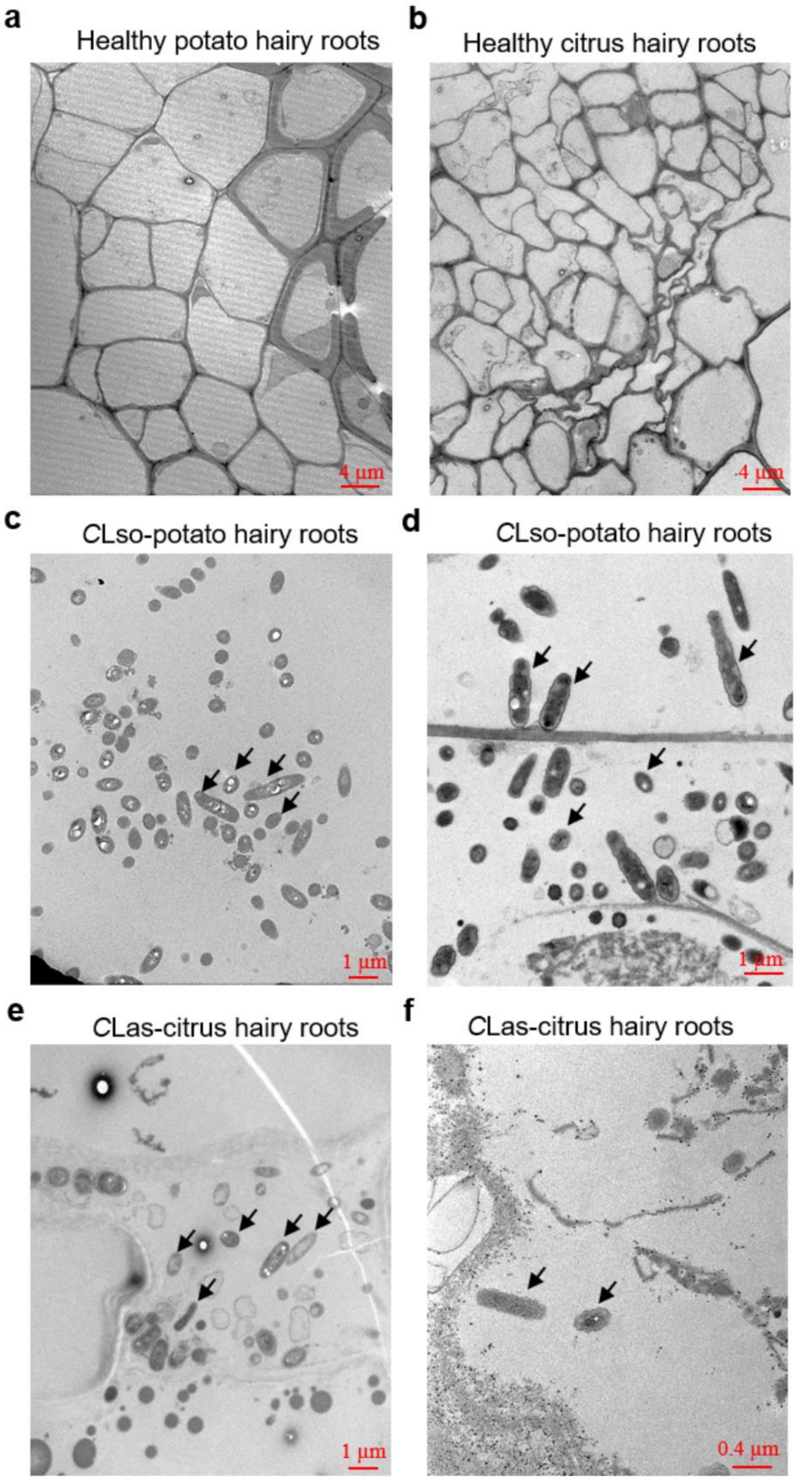

Supplementary Fig. 9. Transmission electron microscopy imaging of *C*Lso-potato and *C*Las-citrus hairy roots. (**a-b**) Cross-section of healthy *C*Lso-potato and *C*Las-citrus hairy roots. Scale bars, 4 µm. (**c-f**) Cross-section of *C*Lso-potato and *C*Las-citrus hairy roots showed multiple round- and bacilliform-shaped bacteria-like cells. Scale bars, 0.4 to 1 µm.

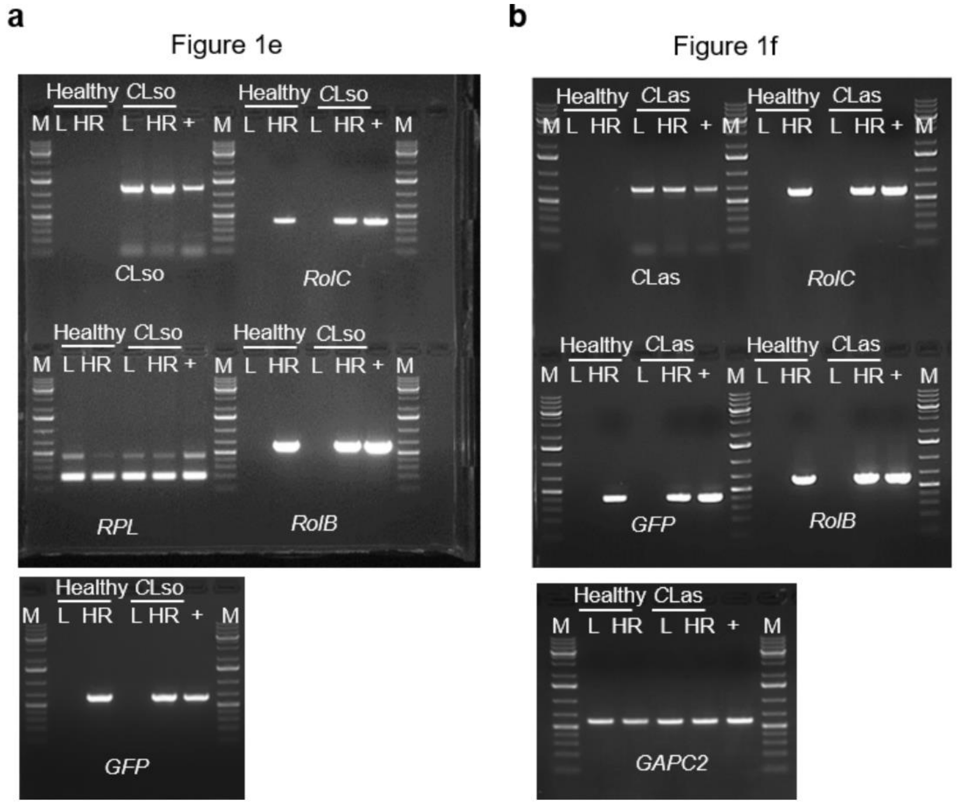

Supplementary Fig. 10. Uncropped raw agarose gel images used to prepare Fig. 1e and 1f.

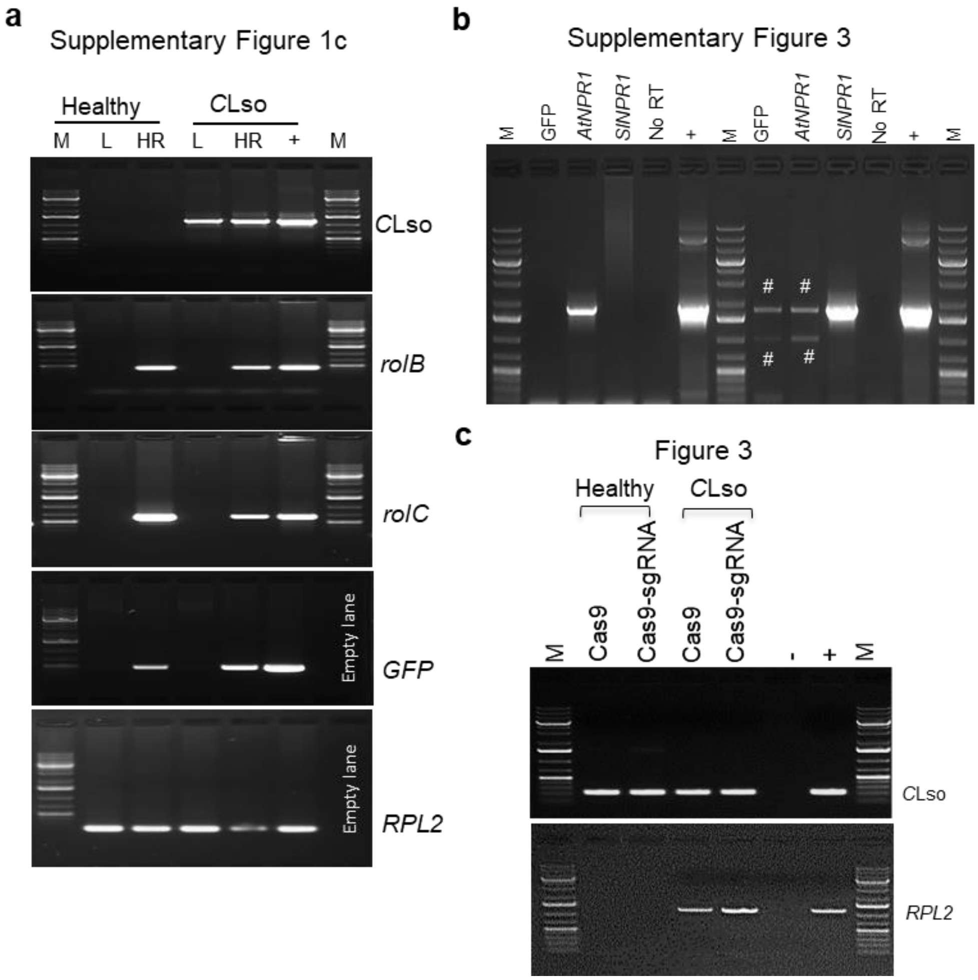

Supplementary Fig. 11. Uncropped raw agarose gel images used to prepare Supplementary Figs. 1c and 3 as well as Fig. 3.

Supplementary References

1. Lin H, *et al.* Genetic diversity of '*Candidatus* Liberibacter solanacearum' strains in the United States and Mexico revealed by simple sequence repeat markers. *Eur. J. Plant Pathol.* **132**, 297-308 (2012).

2. Avila CA, *et al.* Loss of function of *FATTY ACID DESATURASE7* in tomato enhances basal aphid resistance in a salicylate-dependent manner. *Plant Physiol.* **158**, 2028-2041 (2012).

3. Mafra V, *et al.* Reference genes for accurate transcript normalization in citrus genotypes under different experimental conditions. *PLoS ONE* **7**, e31263 (2012).

4. Sengoda VG, Cooper WR, Swisher KD, Henne DC, Munyaneza JE. Latent period and transmission of "*Candidatus* Liberibacter solanacearum" by the potato psyllid *Bactericera cockerelli* (Hemiptera: Triozidae). *PLoS ONE* **9**, e93475 (2014).

5. Levy J, Ravindran A, Gross D, Tamborindeguy C, Pierson E. Translocation of '*Candidatus* Liberibacter solanacearum', the Zebra chip pathogen, in potato and tomato. *Phytopathology* **101**, 1285-1291 (2011).

6. Hocquellet A, Toorawa P, Bove J-M, Garnier M. Detection and identification of the two *Candidatus* Liberibacter species associated with citrus huanglongbing by PCR amplification of ribosomal protein genes of the β operon. *Mol. Cell. Probe* **13**, 373-379 (1999).

7. Zheng Z, Xu M, Bao M, Wu F, Chen J, Deng X. Unusual five copies and dual forms of nrdB in "*Candidatus* Liberibacter asiaticus": Biological implications and PCR detection application. *Sci. Rep.* **6**, 39020 (2016).

8. Triplett BA, Moss SC, Bland JM, Dowd MK. Induction of hairy root cultures from *Gossypium hirsutum* and *Gossypium barbadense* to produce gossypol and related compounds. *In Vitro Cell Dev. Biol. Plant.* **44**, 508-517 (2008).
