# Supplementary material for: Plant hairy roots enable high throughput identification of antimicrobials against Candidatus Liberibacter spp
Source: Nat Commun. 2020 Nov 16;11:5802. doi: 10.1038/s41467-020-19631-x (PMC7669877; doi:10.1038/s41467-020-19631-x)
Supplement: Supplementary file 3 — Reporting Summary [file 41467_2020_19631_MOESM3_ESM.pdf]

nature research

Corresponding author(s): Kranthi Mandadi
Last updated by author(s): 2020/10/21

Reporting Summary

Nature Research wishes to improve the reproducibility of the work that we publish. This form provides structure for consistency and transparency in reporting. For further information on Nature Research policies, see Authors & Referees and the Editorial Policy Checklist.

Statistics

For all statistical analyses, confirm that the following items are present in the figure legend, table legend, main text, or Methods section.

n/a	Confirmed	
☐	☒	The exact sample size (*n*) for each experimental group/condition, given as a discrete number and unit of measurement
☐	☒	A statement on whether measurements were taken from distinct samples or whether the same sample was measured repeatedly
☐	☒	The statistical test(s) used AND whether they are one- or two-sided<br>*Only common tests should be described solely by name; describe more complex techniques in the Methods section.*
☐	☒	A description of all covariates tested
☒	☐	A description of any assumptions or corrections, such as tests of normality and adjustment for multiple comparisons
☐	☒	A full description of the statistical parameters including central tendency (e.g. means) or other basic estimates (e.g. regression coefficient) AND variation (e.g. standard deviation) or associated estimates of uncertainty (e.g. confidence intervals)
☐	☒	For null hypothesis testing, the test statistic (e.g. *F*, *t*, *r*) with confidence intervals, effect sizes, degrees of freedom and *P* value noted<br>*Give P values as exact values whenever suitable.*
☒	☐	For Bayesian analysis, information on the choice of priors and Markov chain Monte Carlo settings
☒	☐	For hierarchical and complex designs, identification of the appropriate level for tests and full reporting of outcomes
☒	☐	Estimates of effect sizes (e.g. Cohen's *d*, Pearson's *r*), indicating how they were calculated

Our web collection on statistics for biologists contains articles on many of the points above.

Software and code

Policy information about availability of computer code

Data collection	No software was used
Data analysis	All data analysis and statistics was performed as described in the figure legends using Microsoft Excel software (version 2009) and Python bioinfokit package (https://github.com/mandadi-lab/bioinfokit).<br><br>For the CRISPR experiments, the single guide RNA (sgRNA) targets were designed using the CRISPR-P design toolset (Lei et al, 2014, Mol. Plant 7, 1494-1496), and the percentage of indels were analyzed using the Inference of CRISPR Edits tool (Hsiau T, et al. 2018, BioRxiv, 251082).<br><br>Identity of Sanger-sequencing products was verified using the nucleotide Basic Local Alignment Search Tool (nBLAST) at NCBI https://blast.ncbi.nlm.nih.gov/Blast.cgi?PROGRAM=blastn&PAGE_TYPE=BlastSearch&LINK_LOC=blasthome<br><br>Phylogenetic analysis was performed using maximum-likelihood method using MEGAX (Kumar et al., 2008, Mol. Biol. Evol. 35, 1547-1549)

For manuscripts utilizing custom algorithms or software that are central to the research but not yet described in published literature, software must be made available to editors/reviewers. We strongly encourage code deposition in a community repository (e.g. GitHub). See the Nature Research guidelines for submitting code & software for further information.

Data

Policy information about availability of data

All manuscripts must include a data availability statement. This statement should provide the following information, where applicable:

- Accession codes, unique identifiers, or web links for publicly available datasets
- A list of figures that have associated raw data
- A description of any restrictions on data availability

The data supporting the findings of this study are available in the Supplementary Information Files. A reporting summary for this article is available as a Supplementary Information file. The datasets and/or plant materials generated and analyzed during the current study are available from the corresponding author upon request. The source data underlying Figs. 1g, 1h, 2d, 2e, 2f, 2g, 4b, 4c, 5b, and Supplementary Figs S4d, S4e, S5, S6a, S6b, S7a, S7b are provided as a Source Data file. The structures of the different chemical compounds were retrieved from the ChemSpider database (http://www.chemspider.com/) (last accessed on 15 October 2020).

Field-specific reporting

Please select the one below that is the best fit for your research. If you are not sure, read the appropriate sections before making your selection.

☒ Life sciences ☐ Behavioural & social sciences ☐ Ecological, evolutionary & environmental sciences

For a reference copy of the document with all sections, see nature.com/documents/nr-reporting-summary-flat.pdf

Life sciences study design

All studies must disclose on these points even when the disclosure is negative.

Sample size	The sample size (3 to 5 biological replicates + 2 technical replicates) used for all experiments was considered sufficient based on prior peer-reviewed studies related to Candidatus Liberibacter spp. pathosystem, as well as our prior experimental trials.<br><br>References:<br>Yang, C., Zhong, Y., Powell, C. A., Doud, M. S., Duan, Y., Huang, Y., & Zhang, M. (2018). Antimicrobial Compounds Effective against Candidatus Liberibacter asiaticus Discovered via Graft-based Assay in Citrus. Scientific reports, 8(1), 17288.<br>Li, J., Pang, Z., Trivedi, P., Zhou, X., Ying, X., Jia, H., & Wang, N. (2017). 'Candidatus Liberibacter asiaticus' encodes a functional salicylic acid (SA) hydroxylase that degrades SA to suppress plant defenses. Molecular Plant-Microbe Interactions, 30(8), 620-630<br>Stover, E., Stange, R. R., McCollum, T. G., Jaynes, J., Irey, M., & Mirkov, E. (2013). Screening antimicrobial peptides in vitro for use in developing transgenic citrus resistant to Huanglongbing and citrus canker. Journal of the American Society for Horticultural Science, 138(2), 142-148.<br>Levy, J., Ravindran, A., Gross, D., Tamborindeguy, C. & Pierson, E. Translocation of 'Candidatus Liberibacter solanacearum', the Zebra Chip Pathogen, in Potato and Tomato. Phytopathology 101, 1285-1291, doi:10.1094/PHYTO-04-11-0121 (2011).
Data exclusions	No data was excluded
Replication	The experiments had three biological replicates comprising of independently treated and processed samples.<br>For all quantitative (q) PCRs, two additional technical replicates were included for each biological replicate sample.<br>For reproducibility, the experiments were repeated independently two to three times and all attempts of replication were successful.<br><br>References:<br>Yang, C., Zhong, Y., Powell, C. A., Doud, M. S., Duan, Y., Huang, Y., & Zhang, M. (2018). Antimicrobial Compounds Effective against Candidatus Liberibacter asiaticus Discovered via Graft-based Assay in Citrus. Scientific reports, 8(1), 17288.<br>Li, J., Pang, Z., Trivedi, P., Zhou, X., Ying, X., Jia, H., & Wang, N. (2017). 'Candidatus Liberibacter asiaticus' encodes a functional salicylic acid (SA) hydroxylase that degrades SA to suppress plant defenses. Molecular Plant-Microbe Interactions, 30(8), 620-630<br>Stover, E., Stange, R. R., McCollum, T. G., Jaynes, J., Irey, M., & Mirkov, E. (2013). Screening antimicrobial peptides in vitro for use in developing transgenic citrus resistant to Huanglongbing and citrus canker. Journal of the American Society for Horticultural Science, 138(2), 142-148.<br>Levy, J., Ravindran, A., Gross, D., Tamborindeguy, C. & Pierson, E. Translocation of 'Candidatus Liberibacter solanacearum', the Zebra Chip Pathogen, in Potato and Tomato. Phytopathology 101, 1285-1291, doi:10.1094/PHYTO-04-11-0121 (2011)
Randomization	There were no experiments that needed randomization other that the tissue samples used for the antimicrobial assays and biological replicates were collected randomly from a given batch of microbial roots/hairy root cultures, that were prior authenticated by molecular diagnostics. The validation and sampling strategy is described in the methods section.
Blinding	The in vitro library screening (Fig. 4) and the AMP screening (Fig. 2g) experiments were blinded. The molecules were given an alias ID (e.g., 1, 2, 3...) in lieu of the actual name. The actual name and chemical structures were subsequently correlated to the sample ID for interpretation.<br><br>The rest of the experiments were not blinded since knowing the identity of the reference gene/antimicrobial (e.g., GFP, NPR1, NPR3, tetracycline) was necessary to optimize the hairy root screening system and to provide proof-of-concept.

Reporting for specific materials, systems and methods

We require information from authors about some types of materials, experimental systems and methods used in many studies. Here, indicate whether each material, system or method listed is relevant to your study. If you are not sure if a list item applies to your research, read the appropriate section before selecting a response.

Materials & experimental systems

n/a	Involved in the study
☒	☐ Antibodies
☒	☐ Eukaryotic cell lines
☒	☐ Palaeontology
☒	☐ Animals and other organisms
☒	☐ Human research participants
☒	☐ Clinical data

Methods

n/a	Involved in the study
☒	☐ ChIP-seq
☒	☐ Flow cytometry
☒	☐ MRI-based neuroimaging
