# Supplementary material for: Plant hairy roots enable high throughput identification of antimicrobials against Candidatus Liberibacter spp
Source: Nat Commun. 2020 Nov 16;11:5802. doi: 10.1038/s41467-020-19631-x (PMC7669877; doi:10.1038/s41467-020-19631-x)
Supplement: Supplementary file 4 — Description of Additional Supplementary Files [file 41467_2020_19631_MOESM4_ESM.pdf]

Description of Additional Supplementary Files

File name: Supplementary Dataset 1

Description: High throughput pre-screening of small-molecules using CLso-potato hairy roots.
