# Supplementary material for: Plant hairy roots enable high throughput identification of antimicrobials against Candidatus Liberibacter spp
Source: Nat Commun. 2020 Nov 16;11:5802. doi: 10.1038/s41467-020-19631-x (PMC7669877; doi:10.1038/s41467-020-19631-x)
Supplement: Supplementary file 2 — Peer Review File [file 41467_2020_19631_MOESM2_ESM.pdf]

Reviewers' comments:

Reviewer #1 (Remarks to the Author):

In this manuscript, Irigoyen et al. developed a clever method for ex vivo culturing Candidatus Liberibacter solanacearum and asiaticus. The authors then applied this method evaluate expression of NPR1 gene, antimicrobial peptides in inhibiting CLso, mutation of NPR3, and screening of antimicrobials. The authors have presented some interesting results, which however are very preliminary and without strong supporting data.
Comments:
1. Figure 1 G&H. The unit for Liberibacter titer is confusing. Please use copy number/gram of tissue. Please indict which represent CLso and which represent CLas.
2. It seems ex vivo culturing Candidatus Liberibacter solanacearum and asiaticus can reach high titers. A Ct value of around 17 was reported here. Please use transmission electron microscopy to confirm the high titer of Candidatus Liberibacter solanacearum and asiaticus.
3. Expression of NPR1 in R. rhizogenes has significant inhibitory effect on CLso compared to the control. The authors need to explain why this is happening by testing the following:
free SA and total SA levels needed to be determined
Expression of multiple immunity related genes
Testing the accumulation of antimicrobials.
4. In addition, for Fig. 2E, the appropriate control should be R. rhizogenes containing the vector without the NPR1 gene to exclude the putative inhibitory effect from R. rhizogenes or the empty vector.
5. For Figs. 2C-D, the appropriate control should be R. rhizogenes containing the vector without the coding sequences for antimicrobial peptides to exclude the putative inhibitory effect from R. rhizogenes or the empty vector. In addition, roots can easily absorb antimicrobial peptides. The authors need to test the inhibitory effect dipping the hairy roots into solutions containing antimicrobial peptides.
6. For the genome editing experiments, the authors seems to generate some chimeric or low efficacy mutations. To make the mutations work in disrupting gene function, the mutation should be biallelic or homozygous. The wild type is more than 50%. It is not convincing that such low efficacy gene editing will have the intended effect. The authors need to test whether NPR3 gene expression is changed, its protein level is changed, whether it has the intended antagonistic effect in suppressing plant defense by testing the expression of immunity related genes.
7. For this in vitro multi-well plat assay, the authors need to conduct minimum Inhibitory Concentration (MIC) and minimum Bactericidal Concentration (MBC) to generate convincing and meaningful results.
8. In the abstract, the authors mentioned a susceptibility gene. If it is referring to NPR3, please check the term susceptibility gene. NPR3 does not qualify as a susceptibility gene based on current nomenclature.

Reviewer #2 (Remarks to the Author):

This manuscript presents an interesting methodology to screen antimicrobial compounds against the non culturable 'Candidatus Liberibacter spp.' pathogens. The manuscript is clearly written and organized and the work is of great interest and potentially could have an impact on this research area. My main criticism is the misleading angle used for this work. The authors keep pushing the idea of "culturing" Liberibacter, when in reality they are not doing this at all. What they are doing is infecting plants with CLso and CLas, something that has been done for many years, and there is plenty of evidence that in the infected plants the bacteria reproduce. I do not understand why the authors want

to present their system as a culturing system. In my opinion the advantage of their system is the high throughput capabilities for checking antimicrobioal compounds. But there is no culturing bacteria here at all. The authors can find many examples of infected plants with these two bacteria, and even there are a few manuscripts where antimicrobial compounds are tested in planta (as examples: Scientific Reports volume 8, Article number: 17288 (2018), https://doi.org/10.1371/journal.pone.0111032). The mentioned papers have the advantage of using grafting and testing the compounds actually in plants, and not only in roots. I see that the advantage and main contributions of the current Irigoyen manuscript is basically in the high throughput capabilities of their system. I suggest changing the title and overall angle of this manuscript to represent what it is about: a high throughput antimicrobial screening system.
1) As mentioned above, the great advantage of this system being high throughput, but has a disadvantage with other system in the fact that these compounds are only tested in roots, and is not clear at all how this will translate when these compounds are applied to fully developed plants in the leaves. One of the highest challenges for these Liberibacter pathogens is the fact that they are limited to the phloem, a niche hard to reach by spraying compounds.
2) Besides the technical advantage of this process presented here, authors should have shown as a proof of principle that at least one of the chemicals they discovered to be antagonistic, actually works in fully developed plants when added (even if only under controlled conditions). In other words, the new systems needs to be validated to have a big impact in the scientific community. Without validation, the impact of this research will be limited. I understand this will take time, but it should be doable to test a few of the microbial compounds in planta in the greenhouse (especially in the potato or tomato system that is faster than citrus)
3) I was surprised for the lack of discussion on root infection by CLas and CLso. There are several papers published by E. Johnson (UF) and other groups in Brazil that studied the root infection of citrus by CLas.
4) In terms of culturing efforts, two manuscripts are missing Schaad et al., 2008; and Parker et al, 2014.
5) Single quotation marks re needed every time they refer to ‘Candidatus Liberibacter spp.’.
6) Fig. 1: G and H: for GE what is the amount of plant material used? How many mg of root material were used? Is hard to make conclusions on multiplication of bacteria if no amount of sample is provided. Please express populations as GE/mg of plant material. Again, the fact that they see a 6x or 3x multiplication of the bacteria in plant material (considering that they used the same amount of root material) does not mean ‘culturing’. They are just seeing plant infection as multiple other people has done for decades.
7) If the authors found the SlNPR1 orthologue in tomato, why is this gene not protecting the plant against CLso infection under natural conditions? Any explanation?
8) Fig. 2: in Fig. 2F AMP7 and AMP8 increase growth of CLso (similarly in Fig. 2 G AMP6 and AMP 8 promote growth of CLas). Any explanation?

Reviewer #3 (Remarks to the Author):

Review of : Plant Hairy Roots Enable ex vivo Culturing 1 of Fastidious Pathogens and Identification of New Antimicrobials

This manuscript is exceptionally well written and describes a marked breakthrough in testing therapeutic approaches for non-culturable plant pathogens. My comments are mainly quite minor, mainly dealing with typos, but I suggest that they merit the authors attention. All my comments below are also highlighted in the text and have associated comment balloons.

The biggest issue is the inconsistency and errors that appear to be abundant in the References. I only read through the first 20, and 6 of them had irregularities. The entire References section should be carefully edited. I did not verify linkage of References and numbers in the manuscript, but suggest this

also be carefully reviewed.

Line 41: t in tuberosum not italicized
Line 48: need to indicate that HLB is haunglongbing and a synonym for citrus greening
Fig 1: last sentence has apparent redundancy
Line 133: italicize Arabidopsis?
Line 180: Likely should be “diverse” rather than “diversity”

Fig. 4: reword 4th line;
in lines 12-14 the numbers in parenthesis should be described as linking to Fig 4 B and C

Line 210: “Academy” not “academy”
Line 212: “for” would be standard, not “to”
Line 234: "gene knockout" would be more accurate
Line 303: Linnaeus did not describe grapefruit. I THINK this should be Macfad.
Line 337: g/molecule? as written could also be g/ mole
Line 370: rephrase. Maybe "all the most-effective compounds" Someone is going to read this as "all the Pb compounds"

Figure titles: as above

Ed Stover, USDA/ARS Ft. Pierce, FL

Irigoyen et al., 2020; Nat. Comm. Response to Reviewer comments:

Reviewer #1 (Remarks to the Author):

In this manuscript, Irigoyen et al. developed a clever method for ex vivo culturing Candidatus Liberibacter solanacearum and asiaticus. The authors then applied this method evaluate expression of NPR1 gene, antimicrobial peptides in inhibiting CLso, mutation of NPR3, and screening of antimicrobials. The authors have presented some interesting results, which however are very preliminary and without strong supporting data.

Comments:

1. Figure 1 G&H. The unit for Liberibacter titer is confusing. Please use copy number/gram of tissue. Please indict which represent CLso and which represent CLas.

We edited the Liberibacter copy number description in the legend. It is now reported as "genome equivalents (GE) per 50 ng of root genomic (g) DNA".

2. It seems ex vivo culturing Candidatus Liberibacter solanacearum and asiaticus can reach high titers. A Ct value of around 17 was reported here. Please use transmission electron microscopy to confirm the high titer of Candidatus Liberibacter solanacearum and asiaticus.

We respectfully clarify that TEM is neither quantitative nor a confirmatory test for CLso and CLas, without doing additional immunolabeling experiments with specific antibodies. In fact, this is a specialized area, and only one or two groups have performed TEM to visualize (not quantify) CLas[1, 2] and no studies exist so far for CLso. To quantify titer, quantitative (q) PCR using primers specific to CLso [3] and CLas [4, 5] are the established assays and are sufficient. We performed additional Sanger DNA sequencing of the PCR amplicons and verified them by BLAST analysis to confirm the sequence identity of CLso and CLas in the respective hairy root cultures. These data have been added to the revised manuscript (Fig. S8). Our copy number analysis indicates that there are ~3000 CLso cells (average Ct=25) and ~12,000 CLas cells (average Ct=19) per 50 ng of root genomic (g) DNA (Fig. 1G–H). These estimates are comparable to those observed in planta [3, 6].

3. Expression of NPR1 in R. rhizogenes has significant inhibitory effect on CLso compared to the control. The authors need to explain why this is happening by testing the following: free SA and total SA levels needed to be determined, Expression of multiple immunity related genes, Testing the accumulation of antimicrobials.

The primary focus of the study was to develop an alternative system to cultivate CLso/CLas in hairy roots and demonstrate its utility for high throughput screening of antimicrobials. Dissecting the mechanism of NPR1 action would deserve to be a stand-alone study. However, as suggested, we did perform additional experiments to demonstrate that functional analysis can be performed using the hairy root system.

1. **First, we measured the expression of three defense-related marker genes (the pathogenesis-related, or PR, genes PR-1 like, PR-3 like and WRKY6-like) in the SlNPR1- and AtNPR1-expressing potato hairy roots, along with empty vector controls in both healthy and CLso-infected conditions. PR gene expression was significantly higher in both SlNPR1- and AtNPR1-expressing hairy roots in healthy conditions, when compared to empty vector controls (Fig. 2E). The induction of PR genes was greatly amplified upon CLso infection in both SlNPR1- and AtNPR1-expressing hairy roots, when compared to vector-alone controls**

(Fig. 2E). Together, these results suggest that SlNPR1 and AtNPR1 function as transcriptional activators of PR genes in potato to mediate defense responses against '*Candidatus* Liberibacter spp.'

2. **Next, we measured the levels of salicylic acid (SA). Interestingly, despite the activation of PR and WRKY defense-related genes, SA levels were significantly lower in SlNPR1- and AtNPR1-expressing hairy roots, in both healthy and CLso-infected conditions, when compared to empty vector controls (Fig. 2F). We propose three scenarios that could explain the lower SA levels in SlNPR1- and AtNPR1-expressing potato hairy roots:**
    a. **SA accumulation in NPR1 overexpressors is directly (positively) associated with the levels of CLso, i.e., lower CLso, lower SA.**
    b. **SA-mediated signaling is far more potent in the NPR1 overexpressors relative to controls, and thus less SA is needed to mediate the defense responses. This hypothesis posits that in empty vector controls, NPR1 concentration/activity is less than optimal for triggering SA-mediated defenses, and so more SA is produced to compensate for relatively lower amounts of NPR1.**
    c. **A negative feedback loop in the NPR1 overexpressors could suppress SA levels in order to maintain defense homeostasis. There is some evidence to support this scenario. Although we did not find previous studies that determined SA levels in NPR1 overexpressing lines, studies of Arabidopsis *npr1* mutants and of other SA biosynthesis mutants indicate that NPR1 participates in negative feedback regulation of SA biosynthesis[7, 8, 9, 10, 11]. Recently, Wang et al. (2020) also showed that there was no concomitant increase in the levels of SA despite activation of multiple SA-related genes (*PR1* and *PAL1*) in maize roots colonized by *Trichoderma*[12]. Thus, our results here support the model that while NPR1 mediates *PR*-gene activation to inhibit *C*Lso in potato, it also negatively regulates SA accumulation to prevent toxic build up and/or to maintain defense homeostasis[7, 8, 9, 10, 11, 12].**

We thank the reviewer for suggesting these experiments, which led to interesting insights into the mechanism whereby NPR1 inhibits '*Candidatus* Liberibacter sp.' and expanded our understanding of the utility of the microbial hairy root system for conducting fundamental studies. We have added these results and discussion to the revised manuscript.

4. In addition, for Fig. 2E, the appropriate control should be R. rhizogenes containing the vector without the NPR1 gene to exclude the putative inhibitory effect from R. rhizogenes or the empty vector.

In all genetic experiments, our controls were indeed hairy roots transformed with the empty vector without a target gene. We clarified this further in the figure legend and the methods.

5. For Figs. 2C-D, the appropriate control should be R. rhizogenes containing the vector without the coding sequences for antimicrobial peptides to exclude the putative inhibitory effect from R. rhizogenes or the empty vector. In addition, roots can easily absorb antimicrobial peptides. The authors need to test the inhibitory effect dipping the hairy roots into solutions containing antimicrobial peptides.

In all genetic experiments, our controls were hairy roots transformed with the empty vector without a target gene. We clarified this further in the figure legend and the methods.

We thank the reviewer for leading us toward a new approach to deliver peptides in the microbial hairy root cultures. Unlike small molecules, peptide uptake/absorption into intact tissues does not occur readily when roots are dipped in a solution. There are several constraints on this process, such as the physical barriers of cell walls, the hydrophobicity of the cell membrane, and size-

exclusion limits, as well as significant proteolytic activity in the apoplast/cell membrane [13, 14, 15, 16, 17]. Several groups are actively working in this area to optimize systemic delivery of proteins into intact plant tissues, but have so far only been successful in protoplasts or when using translational fusions with specific cell-penetrating peptides (CPPs) or carrier cationic peptides, such as a 9-mer polyarginine peptides (R9) [13, 14, 15, 16, 17]. The later tagging approach does come with a risk of disrupting the structure and biological function of the AMP.

However, we explored other ways to deliver smaller peptides into root tissues. For this, we performed vacuum infiltration, in a manner similar to delivery of small molecules *in vitro* (Fig. 4A). As proof of concept, we selected two peptides corresponding to AMP2 and AMP5 and evaluated their use in CLas-citrus hairy root cultures. Both the peptides showed good efficacy in inhibiting CLas and CLso when overexpressed in the microbial hairy roots via T-DNA vectors (Fig. 2G). Briefly, the two peptides were produced recombinantly, and vacuum-infiltrated into the hairy roots at 5 and 10 µg/ml concentration. After 72 h, molecular diagnostics was performed to determine levels of CLas. Both peptides showed statistically significant ($P \leq 0.05$) dose-dependent inhibition of CLas, and the results parallel with those of the genetic-based overexpression assays (Fig. 2G). Although this is yet another useful, and probably faster, approach to test AMPs, it would be challenging to deliver proteins as large as NPR1 or protein complexes such as that required for CRISPR-Cas9. Another caveat to the use of direct protein delivery is the need for appropriate folding/post-translational modifications/native activity of the peptides/proteins when produced by a synthetic or recombinant route. In these situations, genetic-based expression/delivery into plant tissues would be appropriate. Nevertheless, we plan to further explore the upper size limits of proteins that can be delivered by this approach in new studies and thank the reviewer again for leading us into this area of investigation. This extended the *in vitro* assay system for high-throughput screening of AMPs, in a manner similar to that for small molecules. We have added these new results to the revised manuscript (Fig. S5).

6. For the genome editing experiments, the authors seems to generate some chimeric or low efficacy mutations. To make the mutations work in disrupting gene function, the mutation should be biallelic or homozygous. The wild type is more than 50%. It is not convincing that such low efficacy gene editing will have the intended effect. The authors need to test whether NPR3 gene expression is changed, its protein level is changed, whether it has the intended antagonistic effect in suppressing plant defense by testing the expression of immunity related genes.

The main reason for the observed chimeric/low rate (~50%) of mutations in the SlNPR3-experiment (Fig. 3) is the endogenous copy number (ploidy). Potato is tetraploid ($2n = 4x = 48$). This reduces the chances of obtaining biallelic, homozygous mutations, particularly in transient transformation assays, such as with hairy roots in which each root is affected by an independent transformation event. For instance, in CRISPR experiments where we attempted to edit a single-copy GFP transgene, we observed editing rates of ~86–100% (Fig. S3e,f), suggesting that the low rate of mutations seen in editing endogenous genes is inevitable in potato given its polyploidy. Nevertheless, from the perspective of biological significance, even knocking out ~50% of *NPR3* in the hairy root population was sufficient to promote the systemic immune response against CLso, as indicated by the significant decrease in CLso titers in *NPR3*-edited hairy roots when compared to Cas9-alone vector controls (Fig. 3D). Please also note that the intent of this hairy-root-based assay is to expedite testing of loss-of-function of potential targets. If promising results are attained, stable CRISPR lines with the preferred mutations can be generated for further studies, aspects that are beyond the scope of this study. We added the CRISPR discussion points to the revised manuscript.

As for the NPR3 protein/activity, since we do not have antibodies against the endogenous potato NPR3, and to the best of our knowledge there are no other reports of anti-NPR3 antibodies in other systems, we measured the expression of downstream target marker genes (*PR-1 like, PR-3 like*), as well as *WRKY6-like* and *NPR1*, which are transcriptional co-activators in SAR responses. Expression of all four SAR markers was significantly higher in *StNPR3* edited hairy roots than in Cas9 vector control (Fig. 3e), again suggesting that NPR3 activity is sufficiently impaired by the CRISPR editing. We have added these new data to the revised manuscript (Fig. 3e).

7. For this in vitro multi-well plat assay, the authors need to conduct minimum Inhibitory Concentration (MIC) and minimum Bactericidal Concentration (MBC) to generate convincing and meaningful results.

MBC and MIC are defined as the lowest concentration of an antibiotic/chemical that can kill or inhibit target bacteria to a point that there is no growth in culture or plates (>99.9% reduction) as determined visually or by optical density measurements. Unfortunately, it is not technically feasible to estimate MBC and MIC for fastidious pathogens like '*Candidatus* Liberibacter spp.' [18], even in the *ex vivo* hairy root cultures, since they are not axenic cultures. However, one can determine biologically active concentrations by doing dose-response assays in the hairy root system. To demonstrate this, we selected three compounds (#3, #8 and #9) that inhibited both CLso and CLas (Fig. 4) and conducted new dose–response assays with 0, 5, 10, 25 and 50 µM concentrations (Fig. S6). Furthermore, using the dose–response results as a guide, we formulated dosages for subsequent in planta foliar spraying experiments. The results showed good consistency between the hairy root and in planta trials (Fig. 5). These new results are added to the revised manuscript in Fig. S6 and Fig. 5.

8. In the abstract, the authors mentioned a susceptibility gene. If it is referring to NPR3, please check the term susceptibility gene. NPR3 does not qualify as a susceptibility gene based on current nomenclature.

Good point. In the revised manuscript, we referred to this gene as a negative immune regulator.

Reviewer #2 (Remarks to the Author):

This manuscript presents an interesting methodology to screen antimicrobial compounds against the non culturable 'Candidatus Liberibacter spp.' pathogens. The manuscript is clearly written and organized and the work is of great interest and potentially could have an impact on this research area. My main criticism is the misleading angle used for this work. The authors keep pushing the idea of "culturing" Liberibacter, when in reality they are not doing this at all. What they are doing is infecting plants with CLso and CLas, something that has been done for many years, and there is plenty of evidence that in the infected plants the bacteria reproduce. I do not understand why the authors want to present their system as a culturing system. In my opinion the advantage of their system is the high throughput capabilities for checking antimicrobioal compounds. But there is no culturing bacteria here at all. The authors can find many examples of infected plants with these two bacteria, and even there are a few manuscripts where antimicrobial compounds are tested in planta (as examples: Scientific Reports volume 8, Article number: 17288 (2018), https://doi.org/10.1371/journal.pone.0111032). The mentioned papers have the advantage of using grafting and testing the compounds actually in plants, and not only in roots. I see that the advantage and main contributions of the current Irigoyen manuscript is basically in the high throughput capabilities of their system. I suggest changing the title and overall angle of this manuscript to represent what it is about: a high throughput antimicrobial screening system.

We should have better elaborated our rationale for the hairy root 'culturing' approach. It was not our intention to suggest the hairy-root-based culture is akin to monoculture or axenic culture. To provide further context, our original concept of utilizing plant hairy roots as matrices for culturing CLso and CLas was inspired by the classical microbiological techniques developed, and still used, to culture animal and mammalian viruses in host cells, tissues, embryonated eggs, etc. Even though here the host cells are being 'infected', because it was done for the sole purpose of growing and propagating the viruses on demand, the process is referred to as 'culturing' or 'cultivation' [19, 20, 21, 22, 23, 24, 25, 26]. Furthermore, since the above strategies required host tissues removed from the host organism and experimented on or maintained in an external environment, they are classified as *ex vivo* (Latin: "out of the living") approaches.

- ***https://www.cdc.gov/coronavirus/2019-ncov/about/grows-virus-cell-culture.html***
- ***Enders JF, Weller TH, Robbins FC (1949) Cultivation of the Lansing strain of poliomyelitis virus in cultures of various human embryonic tissues. Science 109: 85-87.***
- ***Steinhardt E, Israeli C, Lambert RA (1913) Studies on the cultivation of the virus of vaccinia. The Journal of Infectious Diseases: 294-300.***
- ***Cox HR (1952) Growth of viruses and rickettsiae in the developing chick embryo. Annals of the New York Academy of Sciences 55: 236-247***
- ***Litwin J (1957) A simple method for cultivation of viruses and rickettsiae in the chorio-allantoic ectoderm of the chick embryo by inoculation via the air sac. The Journal of infectious diseases: 100-108***
- ***McClelland L (1946) Simultaneous cultivation of typhus Rickettsiae and Influenza virus in the developing chick embryo. Proceedings of the Society for Experimental Biology and Medicine 63: 427-431.***
- ***Yoshino K (1967) One-day egg culture of animal viruses with special reference to the production of anti-rabies vaccine. Japanese Journal of Medical Science and Biology 20: 111-125.***
- ***Pyrc, K., Sims, A.C., Dijkman, R., Jebbink, M., Long, C., Deming, D., Donaldson, E., Vabret, A., Baric, R., and van der Hoek, L. (2010). Culturing the unculturable: human coronavirus HKU1 infects, replicates, and produces progeny virions in human ciliated airway epithelial cell cultures. J. Virol. 84, 11255-11263.***
- ***Pelzek AJ, Schuch R, Schmitz JE, Fischetti VA (2013) Isolation, culture, and characterization of bacteriophages. Current Protocols Essential Laboratory Techniques 7: 4.4. 1-4.4. 33***

Inspired by the above studies, we formulated our hypothesis that fastidious bacteria such as CLso and CLas are conceptually akin to the obligate viruses, thus *ex vivo* plant (host) tissues would be suitable to culture them in the laboratory. Also, since CLso and CLas are vascular-limited pathogens, we hypothesized that hairy root matrices (with intact vasculature) would be ideal support for their growth. Note: Although we are using the infected plant tissues as source, the produced hairy root cultures are artificially induced by employing *R. rhizogenes* and maintained further in the laboratory. In the revised manuscript, to conform with the terminology of the classical *ex vivo* approaches to cultivate viruses, we replaced culturing with "*ex vivo* cultivation", and added the above rationale/hypothesis in our discussion.

1) As mentioned above, the great advantage of this system being high throughput, but has a disadvantage with other system in the fact that these compounds are only tested in roots, and is not clear at all how this will translate when these compounds are applied to fully developed plants in the leaves. One of the

highest challenges for these Liberibacter pathogens is the fact that they are limited to the phloem, a niche hard to reach by spraying compounds.

We agree with the reviewer that the Liberibacter spp. are hard to reach *in planta* by foliar spraying, as they reside deep in the phloem tissues. As part of any drug-discovery pipeline, the leads will need to be further tested in planta, provided that the delivery systems are improved. The latter is indeed an active area of research, especially in the citrus-HLB community, with several groups evaluating alternative approaches to foliar spraying, such as trunk injections and nanoparticle-based systems [27, 28]. Indeed, the Citrus Disease Sub-committee (CDS) of the National Agricultural Research, Education, Extension and Economics (NAREEE) Advisory Board made "delivery systems for therapeutics, nutrition and other HLB solutions" a #1 priority for the research community to tackle in the FY2020 Emergency Citrus Disease Research and Extension Program. Hopefully, the citrus community will soon find better ways to deliver the active ingredients into citrus trees.

https://nifa.usda.gov/sites/default/files/rfa/FY2020-RFPA-Emergency-Citrus-Preapplication.pdf

As such, the above in planta issues have no bearing on the hairy root bioassays. Conversely, the hairy root system overcomes the in-planta delivery problems in regard to the screening of new compounds, since the compounds and small peptides can be effectively vacuum infiltrated into hairy root tissues. We suggest that hairy root bioassays are thus an ideal pre-screening system for large-scale AI screening and drug discovery pipelines, to narrow down potential new leads before pursuing in planta experiments/trials.

2) Besides the technical advantage of this process presented here, authors should have shown as a proof of principle that at least one of the chemicals they discovered to be antagonistic, actually works in fully developed plants when added (even if only under controlled conditions). In other words, the new systems needs to be validated to have a big impact in the scientific community. Without validation, the impact of this research will be limited. I understand this will take time, but it should be doable to test a few of the microbial compounds in planta in the greenhouse (especially in the potato or tomato system that is faster than citrus)

We agree that it would be useful to compare the results of the hairy root bioassays in planta, but again bear in mind the constraints of in planta delivery as discussed above, especially for citrus. Note that we can already assess this by comparing the efficacy of tetracycline in the hairy root bioassays. Several published reports have established that tetracycline derivatives inhibit CLas in planta when delivered appropriately via trunk injections, but not by foliar spraying[27]. In the hairy root bioassays, we consistently observed that tetracycline significantly inhibited CLas and CLso in hairy roots, thus suggesting that the HR assay data parallels well to the in-planta studies.

Nevertheless, as suggested by the reviewer, we selected three new compounds that showed inhibitory activity against both CLso and CLas (#3, #8 and #9) in the hairy root assays (Fig. 4) and tested them in planta (in potatoes). The three compounds were applied to CLso-infected potatoes by foliar spraying twice a week, at two different dosages (10 µM and 25 µM), and disease symptoms were monitored periodically. Disease progression was monitored for 28 days post infection (dpi), by which point untreated plants showed typical foliar disease symptoms of chlorosis, necrosis, leaf curling and wilting, and were close to dying (Fig. 5a). By contrast, potatoes sprayed with any of the three molecules showed clear tolerance, in a dose-dependent manner, as plants sprayed with 25 µM showed the fewest disease symptoms, on par with those treated with tetracycline (Fig. 5a). The attenuated symptoms were associated with lowered *C*Lso titers in the various treatments, when

compared to untreated controls (Fig. 5B). Together, these experiments demonstrate that the new compounds inhibit '*Candidatus* Liberibacter spp.' in planta, and substantiate the results obtained in the hairy root bioassays. We added the new data and results to the revised manuscript (Fig. 5).

3) I was surprised for the lack of discussion on root infection by CLas and CLso. There are several papers published by E. Johnson (UF) and other groups in Brazil that studied the root infection of citrus by CLas.

We added new discussion on the significance of root biology and root infection in HLB disease development to the revised manuscript.

4) In terms of culturing efforts, two manuscripts are missing Schaad et al., 2008; and Parker et al, 2014.

We added the missing citations to the revised manuscript.

5) Single quotation marks re needed every time they refer to 'Candidatus Liberibacter spp.'.

We edited this throughout the manuscript.

6) Fig. 1: G and H: for GE what is the amount of plant material used? How many mg of root material were used? Is hard to make conclusions on multiplication of bacteria if no amount of sample is provided. Please express populations as GE/mg of plant material. Again, the fact that they see a 6x or 3x multiplication of the bacteria in plant material (considering that they used the same amount of root material) does not mean 'culturing'. They are just seeing plant infection as multiple other people has done for decades.

We edited the copy number description in the legend. It is now reported as "genome equivalents (GE) per 50 ng of root genomic (g) DNA"

7) If the authors found the SlNPR1 orthologue in tomato, why is this gene not protecting the plant against CLso infection under natural conditions? Any explanation?

In this study, we constitutively expressed SlNPR1 in potatoes. Perhaps the endogenous SlNPR1 in tomato does not reach high enough levels/activity or accumulate in a timely manner to robustly inhibit CLso.

8) Fig. 2: in Fig. 2F AMP7 and AMP8 increase growth of CLso (similarly in Fig. 2 G AMP6 and AMP 8 promote growth of CLas). Any explanation?

Good question. We had noticed that too. Given the documented broad-spectrum activities of these AMPs against other bacteria and/or fungi, it is very much possible that in these specific instances, the AMPs could be inhibiting other competitive microbes that are present in the CLso and CLas hairy root cultures.

Reviewer #3 (Remarks to the Author):

Review of : Plant Hairy Roots Enable ex vivo Culturing 1 of Fastidious Pathogens and Identification of New Antimicrobials

This manuscript is exceptionally well written and describes a marked breakthrough in testing therapeutic approaches for non-culturable plant pathogens. My comments are mainly quite minor, mainly dealing

with typos, but I suggest that they merit the authors attention. All my comments below are also highlighted in the text and have associated comment balloons.

Thank you for the positive feedback.

The biggest issue is the inconsistency and errors that appear to be abundant in the References. I only read through the first 20, and 6 of them had irregularities. The entire References section should be carefully edited. I did not verify linkage of References and numbers in the manuscript, but suggest this also be carefully reviewed.

Line 41: t in tuberosum not italicized

Line 48: need to indicate that HLB is haunglongbing and a synonym for citrus greening

Fig 1: last sentence has apparent redundancy

Line 133: italicize Arabidopsis?

Line 180: Likely should be "diverse" rather than "diversity"

Fig. 4: reword 4th line;

in lines 12-14 the numbers in parenthesis should be described as linking to Fig 4 B and C

Line 210: "Academy" not "academy"

Line 212: "for" would be standard, not "to"

Line 234: "gene knockout" would be more accurate

Line 303: Linnaeus did not describe grapefruit. I THINK this should be Macfad.

Line 337: g/molecule? as written could also be g/ mole

Line 370: rephrase. Maybe "all the most-effective compounds" Someone is going to read this as "all the Pb compounds"

Figure titles: as above

Thank you for pointing out these errors. All the suggestions and corrections have been addressed in the revised manuscript.

References

1. Achor D*, et al.* Dynamics of Candidatus Liberibacter asiaticus Movement and Sieve-Pore Plugging in Citrus Sink Cells. *Plant Physiol* **182**, 882-891 (2020).

2. Ammar E-D, Achor D, Levy A. Immuno-Ultrastructural Localization and Putative Multiplication Sites of Huanglongbing Bacterium in Asian Citrus Psyllid Diaphorina citri. *Insects* **10**, 422 (2019).

3. Levy J, Ravindran A, Gross D, Tamborindeguy C, Pierson E. Translocation of '*Candidatus* Liberibacter solanacearum', the Zebra Chip Pathogen, in Potato and Tomato. *Phytopathology* **101**, 1285-1291 (2011).

4. Hocquellet A, Toorawa P, Bove J-M, Garnier M. Detection and identification of the two *Candidatus* Liberibacter species associated with citrus huanglongbing by PCR amplification of ribosomal protein genes of the β operon. *Mol Cell Probes* **13**, 373-379 (1999).

5. Zheng Z, Xu M, Bao M, Wu F, Chen J, Deng X. Unusual five copies and dual forms of nrdB in "*Candidatus* Liberibacter asiaticus": Biological implications and PCR detection application. *Scientific reports* **6**, 39020 (2016).

6. Li W, Hartung JS, Levy L. Quantitative real-time PCR for detection and identification of Candidatus Liberibacter species associated with citrus huanglongbing. *J Microbiol Methods* **66**, 104-115 (2006).

7. Clarke JD, Volko SM, Ledford H, Ausubel FM, Dong X. Roles of salicylic acid, jasmonic acid, and ethylene in cpr-induced resistance in Arabidopsis. *The Plant Cell* **12**, 2175-2190 (2000).

8. Delaney T, Friedrich L, Ryals J. Arabidopsis signal transduction mutant defective in chemically and biologically induced disease resistance. *Proceedings of the National Academy of Sciences* **92**, 6602-6606 (1995).

9. Lu H. Dissection of salicylic acid-mediated defense signaling networks. *Plant signaling & behavior* **4**, 713-717 (2009).

10. Zhang X, Chen S, Mou Z. Nuclear localization of NPR1 is required for regulation of salicylate tolerance, isochorismate synthase 1 expression and salicylate accumulation in Arabidopsis. *J Plant Physiol* **167**, 144-148 (2010).

11. Zhang X*, et al.* Arabidopsis SDG8 Potentiates the Sustainable Transcriptional Induction of the Pathogenesis-Related Genes PR1 and PR2 During Plant Defense Response. *Frontiers in Plant Science* **11**, (2020).

12. Wang K-D, Borrego EJ, Kenerley CM, Kolomiets MV. Oxylipins other than jasmonic acid are xylem-resident signals regulating systemic resistance induced by Trichoderma virens in maize. *The Plant Cell* **32**, 166-185 (2020).

13. Lu S-W*, et al.* Arginine-rich intracellular delivery peptides synchronously deliver covalently and noncovalently linked proteins into plant cells. *J Agric Food Chem* **58**, 2288-2294 (2010).

14. Chang M, Chou J-C, Lee H-J. Cellular internalization of fluorescent proteins via arginine-rich intracellular delivery peptide in plant cells. *Plant Cell Physiol* **46**, 482-488 (2005).

15. Roberts MR. Fast-track applications: The potential for direct delivery of proteins and nucleic acids to plant cells for the discovery of gene function. *Plant Methods* **1**, 12 (2005).

16. Ng KK*, et al.* Intracellular Delivery of Proteins via Fusion Peptides in Intact Plants. *PLOS ONE* **11**, e0154081 (2016).

17. Bilichak A, Luu J, Eudes F. Intracellular delivery of fluorescent protein into viable wheat microspores using cationic peptides. *Front Plant Sci* **6**, 666 (2015).

18. Zhang M, Guo Y, Powell CA, Doud MS, Yang C, Duan Y. Effective antibiotics against '*Candidatus* Liberibacter asiaticus' in HLB-affected citrus plants identified via the graft-based evaluation. *PLoS ONE* **9**, e111032 (2014).

19. Enders JF, Weller TH, Robbins FC. Cultivation of the Lansing strain of poliomyelitis virus in cultures of various human embryonic tissues. *Science* **109**, 85-87 (1949).

20. Steinhardt E, Israeli C, Lambert RA. Studies on the cultivation of the virus of vaccinia. *The Journal of Infectious Diseases*, 294-300 (1913).

21. Cox HR. Growth of viruses and rickettsiae in the developing chick embryo. *Ann N Y Acad Sci* **55**, 236-247 (1952).

22. McClelland L. Simultaneous Cultivation of Typhus Rickettsiae and Influenza Virus in the Developing Chick Embryo. *Proc Soc Exp Biol Med* **63**, 427-431 (1946).

23. Litwin J. A simple method for cultivation of viruses and rickettsiae in the chorio-allantoic ectoderm of the chick embryo by inoculation via the air sac. *The Journal of infectious diseases*, 100-108 (1957).

24. Yoshino K. One-day egg culture of animal viruses with special reference to the production of anti-rabies vaccine. *Japanese Journal of Medical Science and Biology* **20**, 111-125 (1967).

25. Pelzek AJ, Schuch R, Schmitz JE, Fischetti VA. Isolation, culture, and characterization of bacteriophages. *Current Protocols Essential Laboratory Techniques* **7**, 4.4. 1-4.4. 33 (2013).

26. Pyrc K*, et al.* Culturing the unculturable: human coronavirus HKU1 infects, replicates, and produces progeny virions in human ciliated airway epithelial cell cultures. *J Virol* **84**, 11255-11263 (2010).

27. Li J, Pang Z, Duan S, Lee D, Kolbasov V, Wang N. The in planta effective concentration of oxytetracycline against *Candidatus* Liberibacter asiaticus for suppression of citrus Huanglongbing. *Phytopathology*, (2019).

28. Xin X, He Z, Hill MR, Niedz RP, Jiang X, Sumerlin BS. Efficiency of Biodegradable and pH-Responsive Polysuccinimide Nanoparticles (PSI-NPs) as Smart Nanodelivery Systems in Grapefruit: In Vitro Cellular Investigation. *Macromol Biosci* **18**, 1800159 (2018).

Reviewer #1 (Remarks to the Author):

The authors have made tremendous progress in addressing my concerns. I am satisfied with most revisions except the following two:
1. Because the central message of the manuscript is the ex vivo cultivation of Ca. Liberibacter, it is essential for the authors to provide other evidence beyond DNA based detection of Ca. Liberibacter using microscopy-based approach. I think it is doable to observe Ca. Liberibacter under microscopy (TEM or others) with Ct value around 17.
2. It seems to me the genome editing data for GFP is more convincing than the NPR3 data to demonstrate the application of ex vivo cultivation using hairy roots. The argument regarding NPR3 made by the authors are not supported by the low genome editing efficacy and the dramatic effect. But if the authors insist to include the data on NPR3, more supporting data are necessary to show the gene expression of NPR3, protein expression, downstream effect on immune responses and so on in the genome modified lines.

Reviewer #2 (Remarks to the Author):

The authors added several new experiments to their manuscript. It is really impressive to see all the work that was put into this effort, in particular performing the new plant experiments in less than 3 months' time. The work has been improved. But I still strongly disagree with the answer explaining why they are using "ex vivo cultivation" to represent this work. Reading their response letter, it is even clearer that the author are confusing terminology used for virus with the one used with bacteria. All the examples they list in their letter for "cultivation" are all taken from viruses!! The only exception are 2 paper > 70 years old that name Rickettsia. But even in trying to justify this terminology, the authors could not find a single bacterial paper in recent history that uses that terminology. Using "cultivation" is misleading and confusing for bacteriology literature. I understand that they authors say they were "inspired' by work with viruses, which is great, but they need to adjust the terminology to whatever is correct for bacteria. More to the point is that the authors state that "The primary focus of the study was to develop an alternative system to cultivate CLso/CLas in hairy roots and demonstrate its utility for high throughput screening of antimicrobials." I will like the authors to look for any plant pathology paper and find one that says they "cultivated" a prokaryotic pathogen in a plant (even if it is only an organ). In my opinion a more representative title (and therefore focus) of this manuscript will be: "Plant hairy roots enable high throughput identification of new antimicrobials against 'Candidatus Liberibacter spp.' ".

Other comments:
"..reported as "genome equivalents (GE) per 50 ng of root genomic (g) DNA".
How much in plant material weight are 50ng of DNA? Why not use per 1 ng of DNA? Still is hard to compare to other research without knowing how much plant material was used.
The request from another review of TEM is fair. I understand they will not be able to quantify or confirm CLso or CLas with TEM, but if they have lots of cells in the roots (as they think they do), it should be very easy to see them with TEM. And these Liberibacter have very peculiar pleiotropic cell shape, so at least qualitatively they could confirm their presence (further supported by the qPCR data already present).

Reviewer #3 (Remarks to the Author):

Review of : Plant Hairy Roots Enable ex vivo Culturing of Fastidious Pathogens and Identification of New Antimicrobials

This manuscript describes a marked breakthrough in testing therapeutic approaches for non-culturable plant pathogens. My comments are mainly quite minor, mainly dealing with typos, but I suggest that they merit the authors attention. All my comments are highlighted in the text and have associated comment balloons.

It didn't strike me in my initial review, but I don't understand how the genome editing of a GFP transgene (lines 190-199) contributes to this story, since the authors already demonstrated they could edit endogenous genes and affect phenotype. I propose that this be dropped.

In my review of the previous version, I noted inconsistency and errors that appear to be abundant in the References. This was not addressed. The entire References section should be carefully edited. There were six clear errors in the first ten citations, and I stopped there. I did not verify linkage of References and numbers in the manuscript, but suggest this also be carefully reviewed.

I will not indicate here the line by line editorial suggestions. I ask that the authors go through the markup on the manuscript.

Ed Stover

Irigoyen et al., 2020; Nat. Comm. Response to Reviewer comments:

Reviewer #1 (Remarks to the Author):

The authors have made tremendous progress in addressing my concerns. I am satisfied with most revisions except the following two:
1. Because the central message of the manuscript is the ex vivo cultivation of Ca. Liberibacter, it is essential for the authors to provide other evidence beyond DNA based detection of Ca. Liberibacter using microscopy-based approach. I think it is doable to observe Ca. Liberibacter under microscopy (TEM or others) with Ct value around 17.

In the revised manuscript, in collaboration with Amit Levy's group (Univ. of Florida), we performed Transmission Electron Microscopy imaging of the CLso and CLas hairy roots. Multiple round and bacilliform shaped bacteria-like cells[3] were observed in the infected hairy roots, but not in healthy hairy roots (Fig. S9). There were also signs of phloem deterioration, as would be expected from the infected roots[1, 2, 3].

2. It seems to me the genome editing data for GFP is more convincing than the NPR3 data to demonstrate the application of ex vivo cultivation using hairy roots. The argument regarding NPR3 made by the authors are not supported by the low genome editing efficacy and the dramatic effect. But if the authors insist to include the data on NPR3, more supporting data are necessary to show the gene expression of NPR3, protein expression, downstream effect on immune responses and so on in the genome modified lines.

In the revised manuscript, we added expression data for downstream immune marker genes, as well as *NPR3* gene expression. In general, *NPR3* gene expression was induced in CLso-infected hairy roots compared to healthy roots (Fig. 3e). *NPR3* expression was slightly lower in the *NPR3* genome-edited hairy roots, compared to Cas9-transformed hairy roots, but was not statistically significant (Fig. 3e). This was expected, since genome-editing primarily alters coding sequence frames, resulting in protein-level changes not much at the mRNA level. Any effects on mRNA might be either due to edited mRNA being unstable and/or a negative transcriptional feed-back at work. Nevertheless, expression of all the downstream immune response markers (*PR-1 like, PR-3 like, WRKY6-like*), and SAR co-activator *NPR1*, were significantly upregulated in *StNPR3* edited hairy roots compared to Cas9 vector control (Fig. 3e), suggesting that NPR3 functional protein levels or activity was sufficiently impaired in the CRISPR edited hairy roots. The significant reduction in CLso levels in the genome-edited hairy roots (Fig. 3d) correlate well with the enhanced SAR defenses and immune responsive gene activation (Fig. 3e). Together with the GFP transgene editing experiment, we sufficiently demonstrate the feasibility of performing CRISPR-based genome editing using hairy roots.

Reviewer #2 (Remarks to the Author):

The authors added several new experiments to their manuscript. It is really impressive to see all the work that was put into this effort, in particular performing the new plant experiments in less than 3 months' time. The work has been improved. But I still strongly disagree with the answer explaining why they are using "ex vivo cultivation" to represent this work. Reading their response letter, it is even clearer that the author are confusing terminology used for virus with the one used with bacteria. All the examples they list in their letter for "cultivation" are all taken from viruses!! The only exception are 2 paper > 70 years old

that name Rickettsia. But even in trying to justify this terminology, the authors could not find a single bacterial paper in recent history that uses that terminology. Using "cultivation" is misleading and confusing for bacteriology literature. I understand that they authors say they were "inspired' by work with viruses, which is great, but they need to adjust the terminology to whatever is correct for bacteria. More to the point is that the authors state that "The primary focus of the study was to develop an alternative system to cultivate CLso/CLas in hairy roots and demonstrate its utility for high throughput screening of antimicrobials." I will like the authors to look for any plant pathology paper and find one that says they "cultivated" a prokaryotic pathogen in a plant (even if it is only an organ). In my opinion a more representative title (and therefore focus) of this manuscript will be: "Plant hairy roots enable high throughput identification of new antimicrobials against 'Candidatus Liberibacter spp.' .

The reason there were no prior usage of *ex vivo* cultivation terms for plant bacteriology is because this is the first report of using such approach. The closest literature we found were the below two studies that similarly used hairy roots to culture two obligate protozoan parasites:

- Desoignies, N., and Legreve, A. (2011). *In vitro* dual culture of *Polymyxa betae* in *Agrobacterium rhizogenes* transformed sugar beet hairy roots in liquid media. *J. Eukaryot. Microbiol.* 58, 424-425.
- Qu, X., and Christ, B.J. (2007). *In vitro* culture of the obligate parasite *Spongospora subterranea* (Cercozoa; Plasmodiophorida) associated with root□inducing transferred□DNA transformed potato hairy roots. *J. Eukaryot. Microbiol.* 54, 465-467.

Nevertheless, to avoid potential confusion of this approach with conventional axenic mono-culturing, as per the reviewer suggestion, we maintained the focus of the study to high throughput antimicrobial screening and discovery. We also revised the title as suggested by the reviewer: "Plant hairy roots enable high throughput identification of new antimicrobials against '*Candidatus Liberibacter* spp.'

Other comments:
"..reported as "genome equivalents (GE) per 50 ng of root genomic (g) DNA".
How much in plant material weight are 50ng of DNA? Why not use per 1 ng of DNA? Still is hard to compare to other research without knowing how much plant material was used.

In the revised manuscript, as suggested, we expressed the copy number (GE) to 1 ng of gDNA instead. Previously we had it at 50 ng since that was the amount of DNA that was used as template for qPCR assays.

The reason we have not estimated copy number to starting plant material weight is because such reporting comes with potential technical biases. For instance, differences in DNA isolation methodology (and skills) across labs or individuals can yield variable amounts of genomic (g) DNA (quantity and quality) from a given amount of starting tissue, which in turn could affect final copy number estimates. Hence, presenting it relative to amount of DNA extracted[4] allows for comparison across studies regardless of differences in DNA isolation techniques.

- **Levy J, Ravindran A, Gross D, Tamborindeguy C, Pierson E. Translocation of '*Candidatus* Liberibacter solanacearum', the Zebra Chip Pathogen, in Potato and Tomato. *Phytopathology* 101, 1285-1291 (2011).**

The request from another review of TEM is fair. I understand they will not be able to quantify or confirm CLso or CLas with TEM, but if they have lots of cells in the roots (as they think they do), it should be very

easy to see them with TEM. And these Liberibacter have very peculiar pleiotropic cell shape, so at least qualitatively they could confirm their presence (further supported by the qPCR data already present).

In the revised manuscript, we added new Transmission Electron Microscopy imaging data that showed multiple forms of bacteria-like cells (Fig. S9).

Reviewer #3 (Remarks to the Author):

This manuscript describes a marked breakthrough in testing therapeutic approaches for non-culturable plant pathogens. My comments are mainly quite minor, mainly dealing with typos, but I suggest that they merit the authors attention. All my comments are highlighted in the text and have associated comment balloons.
Thank you for the suggestions.

It didn't strike me in my initial review, but I don't understand how the genome editing of a GFP transgene (lines 190-199) contributes to this story, since the authors already demonstrated they could edit endogenous genes and affect phenotype. I propose that this be dropped.
We conducted the GFP transgene CRISPR editing experiments to determine editing rates of a transgene (GFP) vs. endogenous gene which exist as four copies in potatoes, due to tetraploidy. The observed editing rates of GFP transgene were much higher (~86–100%, Fig. S3e,f), compared to the editing rate of endogenous genes (~50%) due to polyploidy. We request to retain this useful information in the manuscript.

In my review of the previous version, I noted inconsistency and errors that appear to be abundant in the References. This was not addressed. The entire References section should be carefully edited. There were six clear errors in the first ten citations, and I stopped there. I did not verify linkage of References and numbers in the manuscript, but suggest this also be carefully reviewed. I will not indicate here the line by line editorial suggestions. I ask that the authors go through the markup on the manuscript.

We apologize for overlooking these errors. In the revised manuscript, we carefully edited the reference section and edited is as per the format of Nat. Comm. and edited all other suggestions in the text.

1. Deng H*, et al.* Phloem regeneration is a mechanism for Huanglongbing-tolerance of "Bearss" lemon and "LB8-9" Sugar Belle® mandarin. *Front. Plant. Sci.* **10**, 277 (2019).

2. Kumar N, Kiran F, Etxeberria E. Huanglongbing-induced anatomical changes in citrus fibrous root orders. *Hort. Sci.* **53**, 829-837 (2018).

3. Achor D*, et al.* Dynamics of '*Candidatus* Liberibacter asiaticus' movement and sieve-pore plugging in citrus sink cells. *Plant Physiol.* **182**, 882-891 (2020).

4. Levy J, Ravindran A, Gross D, Tamborindeguy C, Pierson E. Translocation of '*Candidatus* Liberibacter solanacearum', the Zebra Chip Pathogen, in Potato and Tomato. *Phytopathology* **101**, 1285-1291 (2011).

Reviewer #1 (Remarks to the Author):

2. Regarding NPR3. The authors did not provide the convincing argument to explain the low genome editing efficacy and the dramatic effect. It appears to be the weakest link of the paper. If the authors want to show the application of hairy roots in cultivation of Liberibacters, the authors have the data. IF the authors want to show this in genome editing using NPR3 as an example, the evidence and logic provided do not support their claim. The authors might tune down their claim regarding NPR3. If the authors insist the low genome editing efficacy can reach such results, they can conduct RNAi to silence NPR3 and compare with their genome edited results. The authors need also to test the NPR3 protein level because mRNA level was not changed.

Reviewer #2 (Remarks to the Author):

The authors did a great job improving the manuscript. Regarding the TEM images (Fig. S9), it would have been a better idea to use the lab of Amit Levy's expertise on specific detection of CLas (FISH, antibodies) using fluorescence microscopy to show that the cells are actually CLas and CLso. The lack of bacteria presence in non infected hairy roots of citrus and potato was unexpected. I would have guess that other bacteria (or microorganisms) will be present in the system, there is no reason to anticipate the roots will be sterile. Maybe the authors can explain/discuss this unexpected finding in their paper.

Irigoyen et al., 2020; Nat. Comm. Response to Reviewer comments:

Reviewer #1 (Remarks to the Author):

2. Regarding NPR3. The authors did not provide the convincing argument to explain the low genome editing efficacy and the dramatic effect. It appears to be the weakest link of the paper. If the authors want to show the application of hairy roots in cultivation of Liberibacters, the authors have the data. IF the authors want to show this in genome editing using NPR3 as an example, the evidence and logic provided do not support their claim. The authors might tune down their claim regarding NPR3. If the authors insist the low genome editing efficacy can reach such results, they can conduct RNAi to silence NPR3 and compare with their genome edited results. The authors need also to test the NPR3 protein level because mRNA level was not changed.

Response: We apologize for not clarifying this in the previous revision. In consultation with Michael Kolomiets group (co-author and expert in SAR/plant defense), we included several hypotheses to explain NPR3 editing results. Additionally, since NPR3 is not central to this study, we agree with the reviewer to tone it down. In the revised manuscript, we moved the GFP transgene experiment to the main manuscript as primary example of feasibility of genome editing in hairy roots and moved the NPR3 editing to supplementary section. The following hypotheses are incorporated in the revision:

1. **Systemic SAR activation. Emerging evidence shows that complex antagonistic interactions between NPR3 and NPR1 and other players underpin defense equilibrium in plants[1, 2, 3]. Importantly, the response is systemic, i.e., perpetuated to distant cells and tissues. Thus, in a scenario where NPR3 is edited in some cells/tissues but not in others, the defense response could still be perpetuated to non-edited cells and limit overall pathogen accumulation. This hypothesis is supported by a recent report from Mark Guiltinan lab (Penn State) where they showed that CRISPR-Cas editing of ~27% of *Theobroma cacao* NPR3 copies is sufficient enough to activate defense gene expression and enhance robust resistance to *Phytophthora tropicalis*[4].**
2. **NPR3 dosage sensitivity/haploinsufficiency. Genetic loci often can be in the state of haploinsufficiency or sensitive to dosage, particularly in polyploids[5, 6, 7, 8], i.e., the WT phenotype/trait (i.e., SAR repression) is affected even upon losing a proportion of alleles/copies. It is possible that potato NPR3 responses are dosage dependent[3, 5]. In this scenario, even if a proportion of NPR3 are edited, it could activate SAR.**
3. **NPR3 negative dominance/inhibition. Frameshift SNPs/SNVs, such as those arising from CRISPR editing/mutagenesis of the genome, or post-transcriptional processes like alternative splicing, could result in production of aberrant protein products which lack full functional domains or have altered structures. These truncated/aberrant proteins not only are dysfunctional but create a dominant negative effect by potentially binding and interfering with the native protein complexes[9]. Given the known homodimerization of NPR3[1], it is quite possible that the truncated NPR3 proteins resulting from the edited loci could potentially disrupt the function of the remainder of the native NPR3 and shift the defense homeostasis in favor of SAR activation.**

Reviewer #2 (Remarks to the Author):

The authors did a great job improving the manuscript. Regarding the TEM images (Fig. S9), it would have been a better idea to use the lab of Amit Levy's expertise on specific detection of CLas (FISH, antibodies) using fluorescence microscopy to show that the cells are actually CLas and CLso. The lack of bacteria presence in non infected hairy roots of citrus and potato was unexpected. I would have guess that other bacteria (or microorganisms) will be present in the system, there is no reason to anticipate the roots will be sterile. Maybe the authors can explain/discuss this unexpected finding in their paper.

Response: Thank you. Regarding FISH/antibodies, we did previously check with Dr. Amit Levy for the possibility of doing FISH or Immunogold-EM. In their experience, it is technically very

challenging to perform with plant tissues, particularly for detecting CLas with the current antibodies that are available. So far, Levy group was only successful in performing FISH/immuno-EM to detect CLas in insect-vector[10], not in the plant host. Furthermore, for CLso, there are no antibodies available. Nevertheless, the morphology of the bacteria-like cells we found in the TEM sections of the hairy roots was similar to those observed for CLas previously by Levy's group[11]. In the revised manuscript, we included additional images that illustrate these structures better. Furthermore, since we confirmed both the presence and identity of CLso/CLas in the hairy roots by Sanger DNA-sequencing of CLso/CLas PCR amplicons, it should suffice for the scope of this study. We added these points to the revised manuscript.

Regarding the detection of other microbes in healthy tissue vasculature, we checked with Dr. Levy. In their experience, this is not uncommon, and they do not typically detect bacteria in healthy tissues from greenhouse plants, unless they were infected with some pathogen/fungi or maintained in poor growth conditions. Possible explanation is that unlike diseased samples where a specific pathogen accumulates to high titers sufficient to be detectable by TEM, healthy microbiome of plants could be below the detection limits of TEM. Another reason could be the coverage area. Typical EM sections are quite small (nanometers) and the few sections/areas we observed may not be enough for detecting other microbes that may reside in healthy tissues. Unfortunately, these are the limitations of working with TEM. We added these points to the revised manuscript.

References:

1. Ding Y*, et al.* Opposite roles of salicylic acid receptors NPR1 and NPR3/NPR4 in transcriptional regulation of plant immunity. *Cell* **173**, 1454-1467 (2018).
2. Kuai X, MacLeod BJ, Després C. Integrating data on the Arabidopsis NPR1/NPR3/NPR4 salicylic acid receptors; a differentiating argument. *Front. Plant Sci.* **6**, 235 (2015).
3. Wang W*, et al.* Structural basis of salicylic acid perception by Arabidopsis NPR proteins. *Nature*, 1-6 (2020).
4. Fister AS, Landherr L, Maximova SN, Guiltinan MJ. Transient expression of CRISPR/Cas9 machinery targeting TcNPR3 enhances defense response in Theobroma cacao. *Front. Plant Sci.* **9**, 268 (2018).
5. Veitia RA, Bottani S, Birchler JA. Gene dosage effects: nonlinearities, genetic interactions, and dosage compensation. *Trends Genet.* **29**, 385-393 (2013).
6. Pillitteri LJ, Bemis SM, Shpak ED, Torii KU. Haploinsufficiency after successive loss of signaling reveals a role for ERECTA-family genes in Arabidopsis ovule development. *Development* **134**, 3099-3109 (2007).
7. Morrill SA, Amon A. Why haploinsufficiency persists. *Proc. Natl. Acad. Sci.* **116**, 11866-11871 (2019).
8. Birchler JA, Veitia RA. The gene balance hypothesis: from classical genetics to modern genomics. *Plant Cell* **19**, 395-402 (2007).
9. Herskowitz I. Functional inactivation of genes by dominant negative mutations. *Nature* **329**, 219-222 (1987).
10. Ammar E-D, Achor D, Levy A. Immuno-ultrastructural localization and putative multiplication sites of Huanglongbing bacterium in Asian citrus psyllid *Diaphorina citri*. *Insects* **10**, 422 (2019).
11. Achor D*, et al.* Dynamics of '*Candidatus* Liberibacter asiaticus' movement and sieve-pore plugging in citrus sink cells. *Plant Physiol.* **182**, 882-891 (2020).

Reviewer #1 (Remarks to the Author):

The authors have addressed my concerns.

Reviewer #2 (Remarks to the Author):

No further comments. The authors corrected all the questions satisfactorily.

Irigoyen et al., 2020; Nat. Comm. Response to Reviewer comments:

Reviewer #1 (Remarks to the Author):

The authors have addressed my concerns.

Thank you.

Reviewer #2 (Remarks to the Author):

No further comments. The authors corrected all the questions satisfactorily.

Thank you.
